# ­Identification of QTL for steviol glycoside biosynthesis using a SNP-based genetic linkage map for *Stevia rebaudiana*

**DOI:** 10.1093/g3journal/jkag015

**Published:** 2026-01-20

**Authors:** Keivan Bahmani, Prabhjot Kaur, Nanye Long, Jennifer M Evans, Randolph M Beaudy, Ryan M Warner

**Affiliations:** Department of Horticulture, Michigan State University, East Lansing, MI 48824, United States; Department of Horticulture, Michigan State University, East Lansing, MI 48824, United States; Institute for Cyber-Enabled Research, Michigan State University, East Lansing, MI 48824, United States; Department of Horticulture, Michigan State University, East Lansing, MI 48824, United States; Department of Horticulture, Michigan State University, East Lansing, MI 48824, United States; Department of Horticulture, Michigan State University, East Lansing, MI 48824, United States

**Keywords:** marker-assisted selection, quantitative trait loci, genetic mapping

## Abstract

*Stevia rebaudiana* (stevia) is an herbaceous perennial grown to produce sweet-tasting non-caloric steviol glycosides produced in the leaves and used as a sugar substitute. While stevia produces more than 60 known steviol glycosides, those with the greatest consumer-desired taste profiles, such as rebaudioside (Reb) D and Reb M, are produced at low concentrations. Efforts to breed stevia with increased concentrations of these minor glycosides have been hampered by limited genetic resources to improve breeding efficiency. We developed the first single-nucleotide polymorphism (SNP)-based genetic linkage map for stevia for a highly heterozygous F_1_ population. The linkage map consists of 1322 SNPs across the 11 stevia chromosomes. The map covered 2991.8 cM, although this was inflated by large gaps on linkage group 8. Excluding linkage group 8, the remaining 10 linkage groups covered 1947.7 cM, with an average density of 1.48 cM per marker. The mapping population was grown in multiple locations in 2020 and 2021 to evaluate steviol glycoside production (stevioside and Reb A, B, C, D, E, M, N, and O). The population exhibited transgressive segregation for the production of all evaluated glycosides. QTL were identified for all measured glycosides except Reb M and Reb O. A region of chromosome 1 harbored colocalizing quantitative trait loci (QTL) for stevioside, Reb A, Reb B, Reb D, Reb E, and Reb N. This region contained large-effect QTL explaining up to 38.8% of the observed variation (%VE) for Reb D, and 71.9 and 46.8%VE, respectively, for the minor glycosides Reb E and Reb N. The linkage map and population described herein will be useful for identifying QTL for other stevia growth and yield traits exhibiting quantitative inheritance and will aid in the selection of candidate genes underlying these traits for further evaluation.

## Introduction

*Stevia rebaudiana* (stevia) is a diploid (2n = 22) obligate-outcrossing herbaceous perennial that produces a group of secondary metabolites called steviol glycosides (SGs) that are used in food and beverages as zero glycemic, non-caloric sugar substitutes (Brandle et al. 1998). While the demand for stevia-sweetened products is increasing, the bitter aftertaste associated with the most utilized SGs, including Reb A (Hellfritsch et al. 2012), limits their popularity. Stevia produces at least 64 known SGs (Myint et al. 2020), including compounds, such as Reb D and Reb M, that consumers have identified as having a more desirable taste profile than Reb A (Prakash et al. 2014; Tao and Cho 2020). However, these SGs are produced in much lower concentrations than Reb A (Shafii et al. 2012; Vallejo and Warner 2021). Understanding the genetic basis for the biosynthesis of desired compounds such as Reb D and Reb M will facilitate breeding of new varieties with improved yields of these SGs.

Steviol glycosides are produced primarily in leaf tissues through a series of glycosylation reactions that attach successive sugars, generally glucose but in some cases rhamnose or xylose (Ceunen and Geuns 2013b; Bahmani et al. 2025), to the C-13 and C-19 positions of the backbone aglycone steviol (Brandle et al. 1998). These reactions are catalyzed by UDP-dependent glycosyltransferases (UGTs), including UGT76G1, UGT91D2, and UGT85C2 (Humphrey et al. 2006; Brandle and Telmer 2007; Mohamed et al. 2011; Ceunen and Geuns 2013b; Singh et al. 2017). Reducing expression of *SrUGT76G1*reduced overall production of total SGs (Guleria and Yadav 2013), while overexpressing *SrUGT76G1* increased concentration of Reb A while reducing concentration of the immediate Reb A precursor stevioside (Nasrullah et al. 2023). However, our understanding of the regulation of the SG biosynthetic pathway is incomplete, particularly for consumer-desired SGs such as Reb D and Reb M. QTL analysis of these traits offers the opportunity to identify molecular markers tightly linked to these traits to facilitate breeding for improved concentrations of these metabolites, while also aiding in the identification of causal genes for further functional analysis.

Quantifying steviol glycoside production at the scale necessary for a breeding program is expensive. Developing molecular markers that are tightly linked to SGs of interest would improve the efficiency of breeding. In a previous study of an F_1_ stevia population, the concentrations of most SGs exhibited quantitative inheritance (Vallejo and Warner 2021) and are therefore likely regulated by several genes. Identifying QTL associated with desired SGs offers promise for developing improved varieties and for beginning to elucidate the genetic regulation of their biosynthesis. An initial genetic linkage map for *Stevia rebaudiana* was constructed using random amplified polymorphic DNA (RAPD) markers (Yao et al. 1999). Due to the limited efficiency of RAPD markers, an improved linkage map using simple sequence repeat (SSR) markers, leveraging RNA sequencing data from young fully expanded leaves, shoot apices, flowers, and callus tissues, was developed (Vallejo and Warner 2021). This enhanced map enabled the identification of the first QTL for steviol glycosides, such as Reb D and Reb A, as well as for plant height and vigor, based on phenotypic data from an F_1_ mapping population of 161 individuals (Vallejo and Warner 2021). However, for more precise QTL mapping, a high-density linkage map comprising 11 linkage groups corresponding to the 11 chromosomes of stevia is essential. Genotyping-by-sequencing (GBS)-based single-nucleotide polymorphism (SNP) markers are ideal for developing such a linkage map. These markers can be detected in large quantities through automated processes, are relatively abundant, and offer greater genetic stability compared to SSR markers (Delourme et al. 2013; Tsykun et al. 2017). With the availability of a chromosome-level stevia genome assembly, it is now possible to compare the genetic positions of markers with their physical locations on the stevia chromosomes (Xu et al. 2021).

In this study, we generated SNP markers for a large stevia F_1_ mapping population and employed these markers to develop the first SNP-based genetic linkage map for stevia. This map was then utilized to identify QTL associated with the production of several steviol glycosides. This work provides valuable resources for the development of marker-assisted breeding in stevia and identifies genomic regions of interest to mine for genes regulating SG biosynthesis.

## Materials and methods

### Mapping population development

The MSU18-02 F_1_ mapping population was derived from crossing two selections from the MSU stevia breeding program (10-RJR × 10–19) varying in steviol glycoside profile (Bahmani 2021). The F_1_ seeds were sown in 128-cell trays and grown at the Michigan State University Plant Science Greenhouses at 22 °C and under a 16-h photoperiod (natural photoperiod supplemented with ca. 60 µmol m^−2^ s^−1^ photosynthetically active radiation daily from 0600–2200 Hr). Two hundred thirty-four F_1_ seedlings were randomly selected, established as stock plants, and maintained under the growth conditions described above.

### Genotyping and linkage map generation

DNA was extracted from leaf samples collected from the 234 MSU18-02 F_1_ population individuals and the two parental lines, and these samples were subsequently sent to the University of Minnesota Genomics Center (UMGC) for GBS using the enzyme combination of *MspI* + *BamHI* and employing 150 bp paired-end reads on the NovaSeq 6000 SP platform. Variant calling and genotyping processes were conducted following standard procedures outlined in the Genome Analysis ToolKit (GATK) software suite (DePristo et al. 2011). Prior to variant calling, GBS sequence reads underwent alignment to a reference assembly of the stevia genome (Xu et al. 2021) using the BWA-MEM alignment tool (Li 2013). The reference genome comprised 6978 contigs, with 6358 of these contigs assembled into 11 chromosomes. The finalized chromosome-level genome encompassed 3708 scaffolds, boasting a scaffold N50 value of 106.55 Mb and a cumulative length of 1416 Mb. Following BWA-MEM alignment, reads were subjected to filtering to exclude alignments with a mapping quality (MAPQ) score of less than 20 or those designated as non-primary alignments, thereby eliminating reads that are mapped to multiple locations within the genome.

The resulting alignments were further processed using GATK, and variants were called utilizing its HaplotypeCaller utility. Notably, variant calling was restricted to the 11 chromosomes, despite the reads being mapped to the entire reference assembly, inclusive of unassembled contigs. Variants spanning all F_1_ individuals and the parental lines were combined using the “CombineGVCFs” utility, and genotypes were assigned to everyone using “GenotypeGVCFs”. Subsequently, variants were subjected to filtering, retaining those with a mean sequence depth ≥ 5, variant quality ≥ 50, genotype quality ≥ 10, and missing rate ≤ 0.25. Variants with a minor allele frequency (MAF) < 1% or those harboring more than two alleles were excluded from the dataset, yielding final genotype data for all 236 individuals. Out of the 234 genotyped F_1_ individuals, 221 were used for linkage map analysis, as these lines were planted in at least one of the five field environments. The genotypes, called Variant Call Format (VCF), were converted to a format suitable for JoinMap, wherein alleles in the F_1_ individuals were labeled as missing if they were absent in the parental lines or exhibited Mendelian inheritance errors (i.e. progeny alleles not possible given the parental alleles). Variant sites with an allele missing rate >0.01 underwent filtration, resulting in the generation of a final set of 11,575 loci for constructing the linkage map in JoinMap5 (Van Ooijen 2018).

Pre-mapping filtering involved the removal of markers showing significant segregation distortion (*P* < 0.05), with the number filtered varying across linkage groups (Supplementary Table 1). In addition, markers with a high similarity score (≥0.98) were excluded to prevent inflation of marker density at identical map positions and to simplify map construction. Following these filtering steps, linkage groups were generated using the “Independence LOD’ function, and markers linked under a single LOD threshold were retained for marker ordering. Marker order was determined utilizing the maximum likelihood (ML) method, and map distances were calculated employing the Kosambi mapping function (Kosambi 2016). The final set of markers generated was aligned with their physical positions using AllMaps (Tang et al. 2015) to integrate genetic and physical map information, and markers with conflicting positions were progressively removed. The final map was refined by investigating and removing any remaining redundant markers.

### Phenotyping the mapping population

Twelve three-node tip cuttings from 200 of the MSU18-02 F_1_ lines and the parental lines were taken and rooted in 72-cell trays under intermittent mist. Rooted cuttings were grown in the greenhouse for five weeks before being planted in the field. The mapping population and parents were grown for steviol glycoside evaluation at three sites in 2020: Fort Valley State University (FVSU; Fort Valley, GA), the MSU Horticulture Teaching and Research Center (HTRC) in Holt, MI, and the MSU Southwest Michigan Research and Extension Center (SWMREC) in Benton Harbor, MI. At each location, plantings consisted of raised plastic-covered beds (plastic was white on the outside and black on the inside) with drip irrigation and were arranged in a randomized complete block design with three replications of single-plant plots at each site. In 2021, the mapping population and parents grew as described above at the HTRC and SWMREC locations only. Stock plants were maintained in a greenhouse, and a second round of cuttings was taken and rooted for 2021 field trials. Due to some F_1_ line stock plants dying in the greenhouse during winter 2020–21, some of the original 200 F_1_ lines were not available for 2021 plantings, so additional genotyped MSU18-02 F_1_ lines were included. The number of F_1_ lines for which samples could be collected at each location, planting and sample collection dates, and accumulated growing degree days at each location are listed in Table 1.

**Table 1.** Location information, number of lines planted, planting and sample collection dates, and accumulation of growing degree-days (GDD) for phenotypic evaluation of the MSU18-02 F_1_ genetic mapping population planted at Fort Valley State University (FVSU) in Fort Valley, GA, the MSU horticulture teaching and research center (HTRC) in Holt, MI, and the MSU southwest Michigan research and extension center in Benton Harbor, MI.

Location	Year	Lines planted	Planting date	Sample collection date	GDD
FVSU	2020	196	14 May	10 August	2402.0^a^
HTRC	2020	199	5 June	23 August	1682.9^b^
	2021	178	1 June	25 August	1780.4^b^
SWMREC	2020	192	9 June	26 August	1759.8^b^
	2021	188	3 June	27 August	1816.2^b^

^a^Data from http://www.georgiaweather.net/using 50°F base temperature.

^b^Data from www.enviroweather.msu.edu using 50°F base temperature and Baskerville-Emin method.

At each location/year, 12 weeks after planting, leaf samples, consisting of 10–12 young, fully expanded leaves, were collected from each plant for SG quantification. Leaf samples were dried at 60 °C for 72 h, ground to a fine powder, and 10 mg of dried powder was used for SG extraction. The SG extraction and quantification methods were as previously described (Shafii et al. 2012; Evans et al. 2015). Steviol glycoside quantification was performed on the Waters Acquity TQ-D triple quadrupole mass spectrometer with a Waters Acquity UPLC at the MSU Mass Spectrometry and Metabolomics Core facility. The panel of SGs quantified consisted of stevioside and the rebaudiosides Reb A, B, C, D, E, M, N, and O, and total steviol glycoside (TSG) concentration was determined by adding the concentrations of individual analytes in a sample. To calculate adjusted means for per-environment QTL analysis, a single-environment linear mixed model with genotype as a fixed effect and replicate as a random effect (lmer(Trait ∼ Genotype + (1|Rep), data = data)) was fitted by restricted maximum likelihood (REML) using the lmer() function in the lme4 R package (Smith et al. 2005; Bates et al. 2015). The estimated marginal means were generally similar to the raw means. For broad-sense heritability analysis, variance components were estimated using a linear mixed model with random effects [genotype, environment (defined as the interaction of location and year), genotype-by-environment interaction, and replicate nested within environment] treated as random using the lmer() function in the lme4 R package (model_me < − lmer(Trait ∼ (1|Genotype) + (1|Env) + (1|Genotype:Env) + (1|Env:Rep), data = df, REML = TRUE)). Broad-sense heritability (*H*^2^) was calculated as *H*^2^ = V_G/(V_G + V_GE/n_env + V_e/(n_env × n_rep)) (Getahun et al. 2022), where V_G is the genetic variance, V_GE is the genotype-by-environment interaction variance, V_e is the residual variance, n_env is the number of environments, and n_rep is the mean number of replicates per environment. Pearson correlation coefficients and descriptive statistics were calculated using SPSS v.27 (IBM Corp., Armonk, NY). Pearson correlation coefficients were calculated using raw SG data either within an individual site or with data across all five sites combined.

### QTL identification

MapQTL6.0 (Van Ooijen 2009) was used for identifying QTL underlying biosynthesis of the measured steviol glycosides and TSG. QTL were determined by the interval mapping method (Lander and Botstein 1989; Van Ooijen 1992) using the regression algorithm with default settings. Significant LOD threshold scores for putative QTL were determined for each trait/environment through a 1,000 permutation test at a *P*-value of 0.05. The proportion of variation explained (%VE) by each QTL was estimated by R^2^ values. QTL for the same trait with overlapping intervals of significance across multiple locations/years were considered the same QTL. These robust QTL were visualized using MapChart 2.32 (Voorrips 2002) with aggregate QTL locations defined by 1-LOD drop-off boxes and 2-LOD drop-off whiskers from the peak QTL positions across all significant environments. Haplotypes contributing positive QTL effects were identified for QTL significant across multiple environments by determining haplotype means as described by Klimenko et al. (2010). Briefly, the maternal (10-RJR) genotype was coded as “ab’ and the paternal (10–19) genotype coded as “cd’. MapQTL interval mapping output includes the mean phenotypic values for “ac’, “ad’, “bc’, and “bd’ genotypes. From these, means between “ac’ and “ad’, “bc’ and “bd’, “ac’ and “bc’, and “ad’ and “bd’ provide the haplotype means for “a’, “b’, “c’, and “d’, respectively.

## Results

### SNP marker development

The initial number of raw reads (150 bp paired-end) ranged from 6 million to 36 million pairs across the samples, with a median of 10 million pairs. Post-mapping to the stevia reference genome and subsequent retention of uniquely mapped reads, the count of remaining reads (properly mapped reads) ranged from 6 million to 33 million, with a median of 10 million (individual reads were counted instead of read pairs, considering some reads lacked properly mapped mates). Our finalized genotype data for 236 individuals encompassed 181,614 variant sites, exhibiting an average genotyping rate of 0.74. Subsequently, 181,614 were filtered to eliminate markers with a missing allele rate of 0.01 or higher, resulting in a total of 11,575 SNPs for linkage map development.

### Linkage map generation

Of the 11,575 SNPs, 9,582 (82.7%) were removed prior to mapping due to exhibiting either segregation distortion or similarity to another marker (Supplementary Table 1), leaving 1,993 markers for mapping. Of those, 1,485 markers could be assigned to eleven linkage groups (LGs), corresponding to the eleven stevia chromosomes. After three iterative rounds of AllMaps analysis, markers with conflicting positions were progressively removed, reducing the number from 1,485 to 1,364 and then to 1,355. Supplementary Fig. 2 presents the 1,355 markers retained after the third round of AllMaps integration. An additional 33 redundant markers were subsequently excluded, resulting in a final set of 1,322 markers distributed across 11 LGs (Table 2), covering a cumulative genetic distance of 2,991.8 cM. Although the average marker density across the map was 6.62 cM, it was notably inflated by LG8, which comprised only 18 markers but spanned 1044.1 cM. This group exhibited the lowest marker density at 58.0 cM (Table 2). Notably, approximately 91% of the 841 markers mapped to chromosome 8 displayed segregation distortion and were consequently excluded from the LG8 analysis. Excluding LG8, the remaining ten LGs collectively spanned 1947.7 cM, with individual LGs ranging from 77.1 cM for LG4 to 325.5 cM for LG2 (Table 2). The number of SNPs varied from 67 on LG4 to 209 on LG2. The average marker density across the map was 1.48 cM, with individual LG marker densities, excluding LG8, ranging from 1.15 cM on linkage group 4 to 1.84 cM on LG7. Marker order on the linkage map was largely consistent with physical positions on the reference genome, with some exceptions, most notably a large inversion on LG6 and poor conservation of order on LG8 (Supplementary Fig. 2).

**Table 2.** Summary of linkage map generated by genotyping 221 individuals from the stevia MSU18-02 F_1_ population.

Linkage group	Length (cM)	Number of markers	Average marker density (cM)
1	201.6	152	1.33
2	325.5	209	1.56
3	168.5	122	1.38
4	77.1	67	1.15
5	151.3	89	1.7
6	240.7	171	1.41
7	266.7	145	1.84
8	1044.1	18	58.0
9	172.2	127	1.35
10	159.1	108	1.47
11	185.0	114	1.62
Total	2991.8	1322	6.62

### Steviol glycoside production

The mapping population exhibited transgressive segregation for all measured glycosides and TSG at each location (Table 3; Supplementary Figs. 3–7). SG concentrations were generally higher in 2020 compared to 2021. Within the two Michigan-based sites, mean concentrations of the SGs stevioside, Reb A, Reb D, and Reb M were higher for the population and parents at SWMREC in both years, which corresponded to a greater accumulation of growing degree days at SWMREC during the experimental period (Table 1). Concentrations of several steviol glycosides were significantly correlated. Combining the raw SG data across all five sites, Reb A was correlated positively with stevioside, Reb C, Reb M, and TSG (Table 4). Reb D was correlated negatively with stevioside and Reb A, and correlated positively with Rebs E, M, N, and O. Reb M was correlated negatively with stevioside and Rebs B, C, and E, and correlated positively with Rebs A, N, O, and TSG. Comparing correlations between these pooled data and individual site data for stevioside, Reb A, Reb D, and Reb M revealed largely consistent results (Table 4; Supplementary Table 2). One exception was the correlation between Reb A and Reb M, which were correlated positively for the pooled data and two individual sites (HTRC 2020 and 2021), correlated negatively at FVSU 2020, and were not significantly correlated at SWMREC 2020 and 2021, suggesting a weak correlation between these traits as supported by the relatively small correlation coefficients. Broad-sense heritability estimates were generally high (>0.85) for all SGs except for Reb B, which had an *H*^2^ of 0.476 (Table 5).

**Table 3.** Descriptive statistics for the MSU18-02 stevia mapping population and parents at three locations in 2020 (MSU HTRC, MSU SWMREC, and FVSU) and two (HTRC and SWMREC) in 2021.

Trait	Mapping population MSU18-02					Parental means	
	Number	Minimum	Maximum	Mean^a^	SD	10-19	10-RJR
***HTRC 2020***							
Stevioside (mg/g)	585	2.7	61.97	24.22	14.37	12.69	11.78
Reb A (mg/g)	585	51.33	196.47	110.90	32.72	60.49	148.05
Reb B (mg/g)	585	1.71	12.56	4.50	1.72	2.05	5.85
Reb C (mg/g)	585	3.58	18.76	9.98	3.43	5.1	11.23
Reb D (mg/g)	585	1.44	15.29	6.23	3.06	8.07	5.58
Reb E (mg/g)	585	0.08	1.91	0.64	0.44	0.92	0.18
Reb M (mg/g)	585	0.54	8.22	2.49	1.35	2.36	3.46
Reb N (mg/g)	585	0.5	4.18	1.97	0.76	2.42	1.44
Reb O (mg/g)	585	0.51	6.47	1.72	0.82	1.87	1.84
TSGs (mg/g)	585	77.85	277.94	162.65	43.38	96.01	188.49
***SWMREC 2020***							
Stevioside (mg/g)	536	3.23	63.95	26.49	14.96	14.02	14.99
Reb A (mg/g)	536	71.63	255.92	154.60	41.55	81.87	178.28
Reb B (mg/g)	536	0.28	5.7	1.13	0.59	1.54	2.9
Reb C (mg/g)	536	6.07	23.76	15.46	4.6	7.48	15.93
Reb D (mg/g)	536	2.08	20.83	9.75	4.17	13.38	6.59
Reb E (mg/g)	536	0.06	2.37	0.81	0.57	1.24	0.21
Reb M (mg/g)	536	0.82	18.12	4.97	2.78	5.1	5.47
Reb N (mg/g)	536	0.73	7.17	3.16	1.3	4.13	2.6
Reb O (mg/g)	536	0.3	7.77	2.50	1.43	3.52	2.82
TSGs (mg/g)	536	110.45	318.51	218.87	48.36	132.34	229.82
***FVSU 2020***							
Stevioside (mg/g)	565	5.6	78.93	39.60	20.03	15.91	37.82
Reb A (mg/g)	565	88.27	250.16	148.35	28.83	111.09	178.72
Reb B (mg/g)	565	0	2.71	0.53	0.48	0.53	0.29
Reb C (mg/g)	565	8.61	26.42	17.33	3.97	11.59	18.77
Reb D (mg/g)	565	1.8	23.95	9.19	4.64	16.36	6.5
Reb E (mg/g)	565	0.11	1.96	0.83	0.47	1.41	0.43
Reb M (mg/g)	565	0.5	13.82	4.13	2.85	6.28	3.28
Reb N (mg/g)	565	0.51	8.51	3.22	1.6	6.44	1.71
Reb O (mg/g)	565	0.24	7.4	2.24	1.64	4.42	1.2
TSGs (mg/g)	565	138.4	336.81	225.43	37.2	174.06	248.75
***HTRC 2021***							
Stevioside (mg/g)	515	2.96	64.48	22.69	11.8	14.24	15.41
Reb A (mg/g)	515	21.49	143.60	77.82	19.6	58.42	50.05
Reb B (mg/g)	515	1.32	71.20	13.12	10.6	16.04	9.86
Reb C (mg/g)	515	3.84	38.39	17.64	6.1	10.58	12.36
Reb D (mg/g)	515	0.53	23.32	7.74	4.5	12.11	8.78
Reb E (mg/g)	515	0.02	1.80	0.59	0.4	0.89	0.46
Reb M (mg/g)	515	0.09	7.49	2.11	1.3	2.68	1.94
Reb N (mg/g)	515	0.12	11.94	2.08	1.8	3.80	2.71
Reb O (mg/g)	515	0.12	14.63	2.14	2.2	3.66	3.71
TSGs (mg/g)	515	33.30	232.74	146.00	31.7	122.42	105.29
***SWMREC 2021***							
Stevioside (mg/g)	533	3.94	73.37	26.88	13.0	17.51	16.41
Reb A (mg/g)	533	43.37	132.23	86.38	16.9	61.75	58.3
Reb B (mg/g)	533	0.72	58.76	9.46	10.4	9.54	4.97
Reb C (mg/g)	533	8.68	39.42	22.41	7.3	12.65	15.25
Reb D (mg/g)	533	2.27	26.66	9.40	4.9	17.49	10.17
Reb E (mg/g)	533	0.07	2.03	0.73	0.5	1.21	0.67
Reb M (mg/g)	533	0.54	10.94	3.07	1.6	4.28	2.84
Reb N (mg/g)	533	0.47	7.51	2.37	1.0	2.98	3.01
Reb O (mg/g)	533	0.18	7.92	1.89	1.1	2.81	2.72
TSGs (mg/g)	533	92.2	247.55	162.58	31.9	130.23	114.33

^a^Mixed model-derived adjusted population means.

**Table 4.** Pearson correlation coefficients between stevioside (ST), several rebaudiosides (reb), and total steviol glycosides (TSG) for the stevia F_1_ mapping population MSU18-02 across three field sites in 2020 and two sites in 2021 (*n* = 2768).

	ST	Reb A	Reb B	Reb C	Reb D	Reb E	Reb M	Reb N	Reb O
Reb A	0.308**								
Reb B	−0.148**	−0.298**							
Reb C	0.434**	0.335**	0.304**						
Reb D	−0.070**	−0.065**	−0.026	0.003					
Reb E	0.479**	−0.078**	−0.132**	−0.008	0.502**				
Reb M	−0.447**	0.190**	−0.156**	−0.190**	0.544**	−0.039*			
Reb N	0.109**	0.024	−0.201**	−0.092**	0.520**	0.416**	0.262**		
Reb O	−0.399**	−0.157**	−0.114**	−0.350**	0.451**	0.061**	0.555**	0.673**	
TSG	0.569**	0.921**	−0.132**	0.559**	0.068**	0.132**	0.090**	0.124**	−0.199**

^*^and ** represent significance at *P* < 0.05 and 0.01, respectively.

**Table 5.** Broad-sense heritability (*H*^2^) estimates for steviol glycoside production in the MSU18-02 stevia F_1_ mapping population grown in five environments.

Compound	*H* ^2^
Stevioside (mg/g)	0.978
Reb A (mg/g)	0.880
Reb B (mg/g)	0.476
Reb C (mg/g)	0.932
Reb D (mg/g)	0.956
Reb E (mg/g)	0.971
Reb M (mg/g)	0.941
Reb N (mg/g)	0.915
Reb O (mg/g)	0.943
TSG (mg/g)	0.905

### QTL analysis

**Fig. 1.**
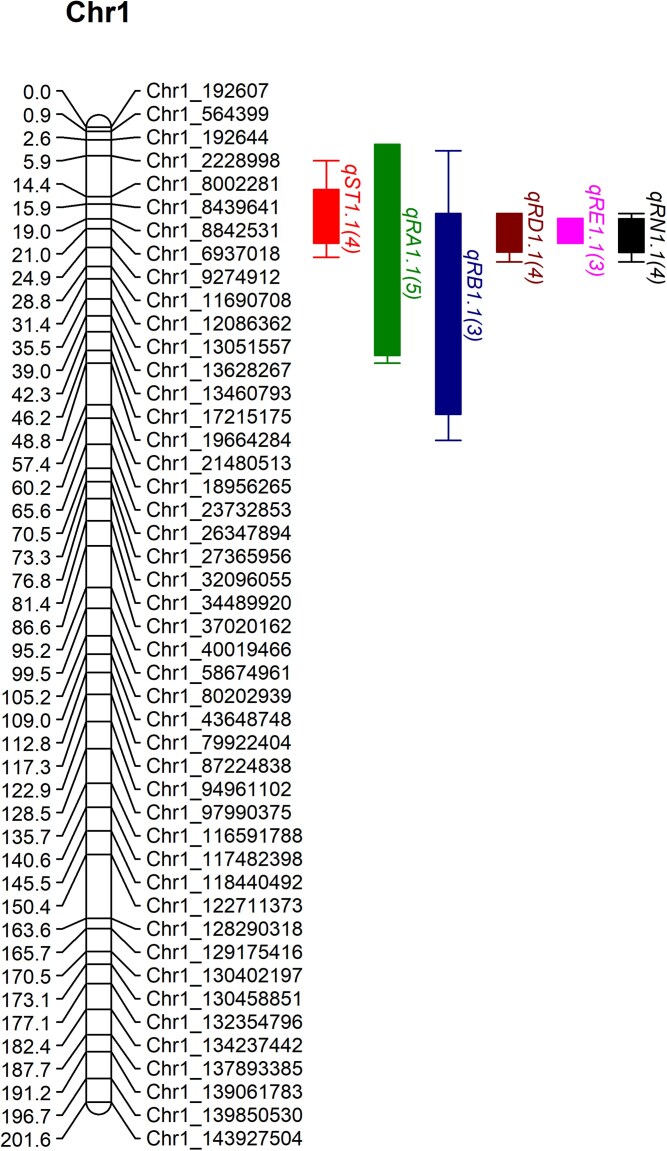
Summary of robust QTL for the steviol glycosides stevioside (*qST*), and rebaudiosides a (*qRA*), B (*qRB*), D (*qRD*), E (*qRE*), and N (*qRN*) on chromosome 1. The boxes and whiskers represent 1-LOD and 2-LOD values, respectively, to either side of the collective peak positions for the QTL. The numbers in parentheses at the end of the QTL name represent the number of environments (out of five) with significant QTL in this region for each trait. Note that only a subset of markers is included on the linkage group to ease visualization.

QTL were identified for all measured glycosides and TSG except for Reb M and Reb O (Table 6; Supplementary Fig. 8) in at least one environment on LGs 1 and 6. QTL with overlapping regions of significance on chromosome 1 (Fig. 1) were identified across at least four of five environments for stevioside (QTL *qST1.1*), Reb A (*qRA1.1*), Reb D (*qRD1.1*), and Reb N (*qRN1.1*). These QTL explained up to 30.2%, 29.3%, 38.8%, and 46.8% of the observed variation for stevioside, Reb A, Reb D, and Reb N, respectively. This same region on chromosome 1 harbored QTL for Reb B (*qRB1.1*) and Reb E (*qRE1.1*) in three of the five environments (Fig. 1), while single environment QTL for Reb D (*qRD1.2*) and Reb N (*qRN1.2*) were located elsewhere on chromosome 1 at FVSU in 2020 (Table 6). Single environment QTL for Reb A (*qRA6.1*), Reb C (*qRC6.1*), and TSG (*qTSG6.1*) were identified on chromosome 6 at FVSU in 2020 and for Reb N *(qRN6.1*) at SWMREC in 2021. Due to low marker number and inflated genetic distances on LG8, QTL on this group were not considered. Positive effect QTL on LG1 were derived from maternal parent 10-RJR (genotype “ab’), but in a haplotype dependent manner (Supplementary Table 3). Specifically, haplotype “a’ contributed positive effects for stevioside, Reb D, Reb E, and Reb N, while haplotype “b’ provided positive effects for Reb A and Reb B. Paternal (10–19) haplotype “c’ and “d’ means were generally similar to the population mean.

**Table 6.** Summary of QTL for the steviol glycosides stevioside (ST), rebaudiosides (reb) a, B, C, D, E, and N, and total steviol glycosides (TSG) across three environments in 2020, and two environments in 2021.

Trait	QTL	LG	Nearest Marker	Environment	Position (cM)	Interval (cM)^a^	LOD	LOD threshold	%VE^b^
**ST**	***qST1.1***	1	Chr1_6937018	FVSU 2020	20.96	17.91–26.89	7	4.3	15.9
		1	Chr1_6937018	SWMREC 2020	20.96	18.91–22.97	14.51	4.3	30.2
		1	Chr1_6937018	HTRC 2020	20.96	18.91–22.97	14.52	4.2	30.2
		1	Chr1_6937018	SWMREC 2021	20.04	6.86–25.89	9.28	4.2	24.4
**Reb A**	***qRA1.1***	1	Chr1_8842531	FVSU 2020	20.04	5.86–32.12	9.62	4.3	21.2
		1	Chr1_8439641	SWMREC 2020	15.92	0–23.97	6.48	4.4	14.8
		1	Chr1_2228998	HTRC 2020	5.86	3.58–10.86^c^	4.72	4.3	11
		1	Chr1_13460793	SWMREC 2021	44.25	21.97–48.81	11.54	4.4	29.3
		1	Chr1_6937018	HTRC 2021	20.96	4.58–47.19^c^	7.82	5.9	21
	*qRA6.1*	6	Chr6_16929693	FVSU 2020	142.07	122.22–192.33^c^	5.69	4.3	13.1
**Reb B**	***qRB1.1***	1	Chr1_8842531	HTRC 2020	19.04	4.89–23.96	14.96	5.2	31
		1	Chr_6937018	SWMREC 2021	21.97	18.09–26.89^c^	6.70	4.8	18.3
		1	Chr1_6937018	HTRC 2021	22.96	20.96–26.88^c^	5.03	4.7	14
	*qRB1.2*	1	Chr1_19664284	SWMREC 2021	52.81	43.25–64.83	7.02	4.8	19
**Reb C**	*qRC6.1*	6	Chr6_19634108	FVSU 2020	143.99	122.22–177.01^c^	5.36	4.3	12.4
**Reb D**	***qRD1.1***	1	Chr1_6937018	SWMREC 2020	22.96	17.91–26.89	18.62	4.2	36.9
		1	Chr1_6937018	HTRC 2020	21.96	17.91–24.89	19.81	4.3	38.8
		1	Chr1_6937018	SWMREC 2021	22.96	18.91–27.89	14.35	4.3	35.1
		1	Chr1_6937018	HTRC 2021	22.96	17.91–26.89	15.01	4.3	36.3
	*qRD1.2*	1	Chr1_17215175	FVSU 2020	47.19	43.25–54.81	10.63	4.2	23.1
**Reb E**	***qRE1.1***	1	Chr1_6937018	FVSU 2020	21.96	20.04–23.97	43.3	4.3	65.8
		1	Chr1_6937018	SWMREC 2020	21.96	19.04–22.97	51.29	4.3	71.9
		1	Chr1_6937018	HTRC 2020	20.04	18.91–22.97	48.36	4.2	69.8
**Reb N**	***qRN1.1***	1	Chr1_6937018	SWMREC 2020	22.96	18.91–24.89	25.47	4.3	46.8
		1	Chr1_6937018	HTRC 2020	22.96	18.91–24.89	24.22	4.4	45.1
		1	Chr1_6937018	SWMREC 2021	22.96	17.91–27.89	11.14	4.3	28.5
		1	Chr1_6937018	HTRC 2021	20.96	20.96–21.96^c^	4.83	4.5	13.5
	*qRN1.2*	1	Chr1_17215175	FVSU 2020	47.19	43.25–55.81	9.45	4.2	20.9
	*qRN6.1*	6	Chr6_60025186	SWMREC 2021	197.63	195.95–201.19	4.81	4.3	13.5
**TSG**	*qTSG6.1*	6	Chr6_19634108	FVSU 2020	143.99	122.22–146.36	6.35	4.3	14.6

QTL listed in bold represents overlapping regions of significance for multiple environments (stable QTL), while non-bolded QTL represents single-environment QTL.

^a^Interval indicates the 2-LOD value drop-off region from the peak position.

^b^%VE - Percentage of observed variation explained by the QTL.

^c^For these QTL, the peak position was less than 2-LOD above the threshold LOD, so the reported interval is the region above the threshold LOD value.

## Discussion

Improved understanding of the genetic basis of steviol glycoside production could facilitate development of varieties with increased production of SGs with a consumer-desired taste profile, such as Reb D (Tao and Cho 2020), as genotypes vary widely in SG profile (Ahmad et al. 2020). To achieve this goal, a high-density genetic linkage map is essential for precisely mapping these traits and facilitating marker-assisted selection breeding strategies. Previous efforts utilized linkage maps based RAPD and inter simple sequence repeat (ISSR) markers to assess genetic diversity in stevia germplasm panels (Yao et al. 1999; Heikal et al. 2008; Chester et al. 2013). However, due to their limited reproducibility, these are of limited value for marker-assisted selection. A more recent linkage map based on co-dominant SSR markers covered a distance of 582 cM across 13 linkage groups (Vallejo and Warner 2021). Nonetheless, the efficiency and small marker numbers of SSR markers pose constraints on constructing a high-resolution genetic map. SNPs offer numerous advantages for genetic mapping, including abundance, co-dominance, reproducibility, and lack of associated phenotype (Ganal et al. 2009).

Here, we present a novel stevia linkage map constructed from 1322 SNP markers condensed into 11 linkage groups, corresponding to the 11 chromosomes of the stevia genome (Xu et al. 2021). Notably, this map exhibits a markedly higher average marker density compared to previously published maps, with an average spacing of 1.48 cM, in contrast to 6.0 cM and 7.6 cM in earlier studies (Yao et al. 1999; Vallejo and Warner 2021). Despite this improvement, regions of low marker density occur across several LGs, and some (e.g. LGs 1, 2, 6, and 7) are somewhat inflated in length (>200 cM). A potential explanation for low marker density regions is the abundance of repetitive elements in stevia, which comprise 80.11% of the stevia reference genome assembly (Xu et al. 2021), with 65% of the genome comprised of long terminal repeat retrotransposons (LTR-RTs). For example, the region from ca. 94–120 cM on LG2 exhibits reduced marker density (Supplementary Fig. 1). This region maps to the physical location of ca. 50–90 Mb on chromosome 2 of the reference genome (Supplementary Fig. 2), which corresponds to the region of highest density of *Copia*-type LTR-RTs on that chromosome (Xu et al. 2021). Similar associations were observed for low marker density regions of LGs6 (74–100 cM) and 11 (10–66 cM). A potential explanation for inflated linkage group length is the observation of clusters of highly similar (similarity ≥ 0.98) markers that were removed during pre-mapping filtering. For example, approximately 58 and 70% markers were removed due to similarity on LGs 1 and 2, respectively. Although this level of inflation is common, keeping only a subset of these markers may cause uneven spacing, potentially inflating map distances in certain regions. Additionally, genotyping errors, commonly observed at higher frequencies in GBS than other genotyping approaches (Montanari et al. 2019), may also factor in the observed genetic distance inflation of some linkage groups. Nonetheless, these linkage groups have a relatively even distribution of markers, and the QTLs identified on them remain robust.

While marker order between the linkage map and the reference genome was generally conserved, several variations were identified, including a large inversion on LG6 (Supplementary Fig. 2). Structural and numerical differences in chromosomes have been observed in the genus *Stevia* (Frederico et al. 1996). Frederico et al. (1996) identified widespread inversions among South American *Stevia* spp. and concluded that pericentric inversions were likely a primary mechanism of evolution in the genus. While we believe the current study is the first report of such an inversion among *S. rebaudiana* germplasm, discussions with breeders suggest that this is not uncommon in the species (R.W. personal communication). Numerical chromosomal variation in the forms of aneuploidy and polyploidy also naturally occur within the genus (Frederico et al. 1996). Additionally, an induced-polyploid stevia cultivar exhibiting aneuploidy was recently developed (Markosyan et al. 2021), suggesting that *S. rebaudiana* may be tolerant of aneuploidy. Integration of the genetic and physical map information in AllMaps to remove markers with conflicting positions resulted in removal of ca. 10% of the markers on LGs 2 and 7, and close to 20% of markers on LGs 3 and 11 (Supplementary Table 1). Given the observed structural variations described above, it is possible that these removed markers may represent some additional structural variation between the germplasm described herein and the reference genome genotype.

Linkage group 8 is notably long at 1,044 cM but contains only 18 markers. Most markers on this LG showed significant segregation distortion, with 91% removed during pre-mapping filtering, leaving only a few reliable markers for mapping. This low marker density reduces the confidence in determining marker order, as a small number of markers cannot accurately reflect recombination patterns. All SNPs used in the map aligned uniquely, confirming their reliability, which suggests that LG8's unusual mapping behavior is likely due to biological and genomic features rather than technical alignment errors. The high repetitive element content and duplicated regions in the stevia genome (Xu et al. 2021) can indirectly affect recombination by promoting mispairing or unequal crossing over during meiosis, leading to inflated genetic distances, large gaps, and unlinked markers through chromosomal variations such as aneuploidy, structural variations, and suppressed recombination within repetitive or duplicated regions. Recombination fraction and LOD heatmaps (Supplementary Fig. 9) confirmed that many markers on LG8 were unlinked. Additionally, some of the LG8 markers show higher than expected recombination frequency with markers on LGs 1, 6, 7, and 11. Due to these challenges, LG8 is less reliable for QTL mapping, and any QTLs assigned to this region in the current study were not considered. Approaches to investigate the potential causes of LG8 map inflation include (1) increasing marker density to improve coverage and reduce large gaps, (2) validating a subset of markers with independent genotyping methods such as KASP or Sanger sequencing to verify genotype accuracy and ensure marker reliability, (3) analyzing sequencing read depth to detect possible copy-number variations that might indicate missing or extra chromosome segments. Additionally, reconstructing LG8 in a different mapping population (another cross) would help determine whether the observed map distortion is cross-specific or biologically inherent. If suppressed recombination and distortion on LG8 are consistently reproduced in independent populations, it would strongly suggest a biological basis.

Similarly, on chromosome 4 (LG4), approximately 75% of markers were removed during pre-mapping filtering due to strong segregation distortion, leaving only a small number of reliable markers. The biological and genomic factors discussed above likely also contribute to the partial coverage observed on LG4 (Supplementary Fig. 2). However, the retained markers were sufficient to generate a local linkage map for this region, and any QTLs identified in this region are considered high confidence for downstream analyses.

Concentrations of TSG and individual glycosides varied by location and year (Table 3), with FVSU 2020 producing the highest TSG values. This is consistent with previous results showing that environment influences SG concentrations, though the relative proportions of individual SGs are largely consistent across environments (Barbet-Massin et al. 2016). Among environmental variables, several aspects of the light environment, including photoperiod (Metivier and Viana 1979; Ceunen and Geuns 2013a), light quality (Ceunen et al. 2012), and total daily radiation, or daily light integral (DLI; Evans et al. 2015), have all been shown to influence SG concentrations. Long-day photoperiods generally increase SG production compared to short-day conditions (Ceunen and Geuns 2013a). Stevia is a facultative short-day plant (Warner 2022), so long photoperiods promote vegetative growth. Studies evaluating the impact of different “long-day” photoperiods on SG production are limited, but increasing photoperiod from 15 to 16 h increased leaf concentrations of stevioside and Reb A (the only SGs evaluated) after 4-to-16-day exposures to those photoperiods (Andrade et al. 2023). Compared to the FVSU location, the field locations in Michigan (HTRC and SWMREC) receive a longer photoperiod, but a lower DLI (Faust and Logan 2018) during the experimental period. A previous evaluation of two stevia genotypes indicated that TSG concentration increased as DLI increased from 3.55 to 8.53 mol m^−2^ d^−1^, but was similar with further increases in DLI up to 39.7 mol m^−2^ d^−1^ (Evans et al. 2015). Information is lacking on how temperature and temperature accumulation over time impact SG concentrations. In the current study, within a year, mean TSG concentration increased as growing degree-day accumulation during the experimental period increased (Tables 1 and 3), suggesting that the influence of temperature on leaf concentrations of SGs warrants further investigation.

Consistent with previous results (Vallejo and Warner 2021), all measured SGs exhibited transgressive segregation at each location (Table 3; Supplementary Figs. 3–7). Individual SGs generally exhibited either normal or bimodal population distributions, suggesting that production of individual SGs and TSG is quantitatively inherited. Consistent with this, QTL were identified for all measured SGs except for Reb M and Reb O (Table 6). The QTL region underlying stevioside and Rebs A, B, D, E, and N on LG1 co-localizes with the glycosyltransferase gene *SrUGT76G1* (Xu et al. 2021; gene model Streb.1G003400; starting position Chr1:6,203,060), which has been previously identified as a major regulator of the production of several SGs, including Reb A (Mohamed et al. 2011; Madhav et al. 2013; Olsson et al. 2016; Petit et al. 2019) and a cluster of four genes encoding *ent*-kaurenoic acid hydroxylases (KAH) [Streb.1G007430 (Chr1:14,489,619), Streb.1G007460 (Chr1: 14,545,847), Steb.1G007470 (Chr1:14,574,009), and Streb.1G007480 (Chr1:14,641,684)], which catalyze the conversion of *ent-*kaurenoic acid to steviol, the first committed step in the SG biosynthetic pathway (Brandle and Telmer 2007). These results highlight the utility of the genetic linkage map and mapping population described herein to identify genomic regions associated with the production of SGs of interest and suggest that these resources will be useful for identifying marker-trait associations for other quantitative traits of importance in stevia. This result also suggests that this chromosomal region is of interest for further investigation, as it may harbor other genes important for the regulation of SG biosynthesis.

Stevia presents several challenges to breeders (Sanabria-Velazquez et al. 2025), including self-incompatibility (Yadav et al. 2011), poor seed longevity (Shuping and Shizen 1995), and considerable expense of quantifying steviol glycosides at the scale of a breeding program. As such, breeding efforts have largely focused on developing improved populations or synthetic cultivars (Warner et al. 2022) instead of elite genotypes producing high concentrations of desired SGs. The development of a SNP-based genetic linkage map anchored to a chromosome-scale genome assembly for stevia will facilitate the identification of molecular markers tightly linked to the production of specific SGs and improve the cost efficiency of breeding for a particular SG profile. Additionally, these resources will aid in the identification of causal genes underlying SG production and other traits of interest in stevia.

## Supplementary Material

jkag015_Supplementary_Data

## Data Availability

The raw sequence data for marker development have been deposited in the NCBI Sequence Read Archive under BioProject ID PRJNA1262366. Mapping population genotyping and phenotyping data, and QTL analysis results, are available at FigShare (https://doi.org/10.25387/g3.30988111). Supplemental material available at G3 online.
