# Supplementary material for: ­Identification of QTL for steviol glycoside biosynthesis using a SNP-based genetic linkage map for Stevia rebaudiana
Source: G3 (Bethesda). 2026 Jan 20;16(3):jkag015. doi: 10.1093/g3journal/jkag015 (PMC12958801; doi:10.1093/g3journal/jkag015)

**Supplementary Information**

**Supplementary Table S1.** Summary of SNP marker development for linkage map generation, including the initial number of markers mapping to each chromosome of the reference genome, and the number of markers removed at each filtering step prior to final map construction.

| **Linkage Group** | **Initial markers (no.)** | **Markers removed for segregation distortion or similarity (no.)** | **Markers retained for mapping by LOD threshold method (no.)** | **Markers per LG after ML mapping (no.)** | **Markers removed in All Maps steps (no.)** | **Markers manually removed due to redundancy (no.)** | **Final markers per LG (no.)** |
| --- | --- | --- | --- | --- | --- | --- | --- |
| **1** | 960 | 738 | 222 | 156 | 4 | 0 | 152 |
| **2** | 1520 | 1209 | 311 | 237 | 24 | 4 | 209 |
| **3** | 803 | 617 | 186 | 159 | 31 | 6 | 122 |
| **4** | 1035 | 922 | 113 | 67 | 0 | 0 | 67 |
| **5** | 858 | 710 | 148 | 97 | 4 | 4 | 89 |
| **6** | 1538 | 1288 | 250 | 187 | 9 | 7 | 171 |
| **7** | 1149 | 952 | 197 | 165 | 17 | 3 | 145 |
| **8** | 841 | 797 | 44 | 18 | 0 | 0 | 18 |
| **9** | 1108 | 931 | 177 | 141 | 9 | 5 | 127 |
| **10** | 823 | 663 | 160 | 117 | 8 | 1 | 108 |
| **11** | 940 | 755 | 185 | 141 | 24 | 3 | 114 |
| **Total** | 11575 | 9582 | 1993 | 1485 | 130 | 33 | 1322 |

**Supplementary Table S2**. Pearson correlation coefficients between steviol glycosides for an F_1_ mapping population evaluated across five sites.

| **FVSU 2020** | | | | | | | | | |
| --- | --- | --- | --- | --- | --- | --- | --- | --- | --- |
|  | **ST** | **Reb A** | **Reb B** | **Reb C** | **Reb D** | **Reb E** | **Reb M** | **Reb N** | **Reb O** |
| **Reb A** | .318^**^ |  |  |  |  |  |  |  |  |
| **Reb B** | -.291^**^ | -.172^**^ |  |  |  |  |  |  |  |
| **Reb C** | .675^**^ | .830^**^ | -.225^**^ |  |  |  |  |  |  |
| **Reb D** | -.352^**^ | -.326^**^ | .198^**^ | -.384^**^ |  |  |  |  |  |
| **Reb E** | .474^**^ | -.183^**^ | -0.058 | .119^**^ | .546^**^ |  |  |  |  |
| **Reb M** | -.740^**^ | -.241^**^ | .268^**^ | -.549^**^ | .693^**^ | -.102^*^ |  |  |  |
| **Reb N** | -.245^**^ | -.187^**^ | 0.019 | -.260^**^ | .859^**^ | .535^**^ | .589^**^ |  |  |
| **Reb O** | -.625^**^ | -.254^**^ | .132^**^ | -.510^**^ | .734^**^ | 0.065 | .880^**^ | .794^**^ |  |
| **TSG** | .655^**^ | .899^**^ | -.226^**^ | .922^**^ | -.219^**^ | .189^**^ | -.380^**^ | -0.070 | -.322^**^ |
|  |  |  |  |  |  |  |  |  |  |
| **HTRC 2020** | | | | | | | | | |
|  | **ST** | **Reb A** | **Reb B** | **Reb C** | **Reb D** | **Reb E** | **Reb M** | **Reb N** | **Reb O** |
| **Reb A** | .361^**^ |  |  |  |  |  |  |  |  |
| **Reb B** | .177^**^ | .753^**^ |  |  |  |  |  |  |  |
| **Reb C** | .659^**^ | .918^**^ | .690^**^ |  |  |  |  |  |  |
| **Reb D** | 0.073 | -0.005 | -.357^**^ | 0.020 |  |  |  |  |  |
| **Reb E** | .524^**^ | -.100^*^ | -.418^**^ | .120^**^ | .734^**^ |  |  |  |  |
| **Reb M** | -.415^**^ | .083^*^ | -0.064 | -.114^**^ | .546^**^ | -0.019 |  |  |  |
| **Reb N** | .179^**^ | -.234^**^ | -.335^**^ | -.105^*^ | .610^**^ | .724^**^ | .113^**^ |  |  |
| **Reb O** | -.476^**^ | -.438^**^ | -.358^**^ | -.529^**^ | .440^**^ | .134^**^ | .525^**^ | .547^**^ |  |
| **TSG** | .604^**^ | .954^**^ | .671^**^ | .977^**^ | .119^**^ | .139^**^ | 0.020 | -0.070 | -.446^**^ |
|  |  |  |  |  |  |  |  |  |  |
| **SWMREC 2020** | | | | | | | | | |
|  | **ST** | **Reb A** | **Reb B** | **Reb C** | **Reb D** | **Reb E** | **Reb M** | **Reb N** | **Reb O** |
| **Reb A** | .251^**^ |  |  |  |  |  |  |  |  |
| **Reb B** | -.094^*^ | -.115^**^ |  |  |  |  |  |  |  |
| **Reb C** | .545^**^ | .907^**^ | -.151^**^ |  |  |  |  |  |  |
| **Reb D** | -0.068 | -.232^**^ | 0.050 | -.141^**^ |  |  |  |  |  |
| **Reb E** | .457^**^ | -.265^**^ | 0.007 | -0.013 | .716^**^ |  |  |  |  |
| **Reb M** | -.554^**^ | -0.016 | 0.077 | -.160^**^ | .514^**^ | -.118^**^ |  |  |  |
| **Reb N** | .107^*^ | -.390^**^ | .111^**^ | -.315^**^ | .445^**^ | .668^**^ | -.104^*^ |  |  |
| **Reb O** | -.569^**^ | -.500^**^ | .218^**^ | -.626^**^ | .360^**^ | 0.073 | .482^**^ | .533^**^ |  |
| **TSG** | .493^**^ | .951^**^ | -.104^*^ | .963^**^ | -0.073 | -0.008 | -0.062 | -.245^**^ | -.531^**^ |
|  |  |  |  |  |  |  |  |  |  |
| **HTRC 2021** | | | | | | | | | |
|  | **ST** | **Reb A** | **Reb B** | **Reb C** | **Reb D** | **Reb E** | **Reb M** | **Reb N** | **Reb O** |
| **Reb A** | -0.034 |  |  |  |  |  |  |  |  |
| **Reb B** | -0.033 | .149^**^ |  |  |  |  |  |  |  |
| **Reb C** | .358^**^ | .842^**^ | .226^**^ |  |  |  |  |  |  |
| **Reb D** | -0.058 | -0.056 | 0.031 | -0.041 |  |  |  |  |  |
| **Reb E** | .544^**^ | -.295^**^ | -0.044 | -.106^*^ | .265^**^ |  |  |  |  |
| **Reb M** | -.607^**^ | .092^*^ | 0.042 | -.249^**^ | .550^**^ | -.096^*^ |  |  |  |
| **Reb N** | .234^**^ | -.244^**^ | -0.028 | -.188^**^ | .104^*^ | .100^*^ | -.119^**^ |  |  |
| **Reb O** | -.181^**^ | -.255^**^ | -.127^**^ | -.401^**^ | .271^**^ | 0.012 | .321^**^ | .717^**^ |  |
| **TSG** | .420^**^ | .791^**^ | .451^**^ | .891^**^ | .141^**^ | 0.063 | -.086^*^ | 0.014 | -.191^**^ |
|  |  |  |  |  |  |  |  |  |  |
| **SWMREC 2021** | | | | | | | | | |
|  | **ST** | **Reb A** | **Reb B** | **Reb C** | **Reb D** | **Reb E** | **Reb M** | **Reb N** | **Reb O** |
| **Reb A** | -0.006 |  |  |  |  |  |  |  |  |
| **Reb B** | -0.069 | .313^**^ |  |  |  |  |  |  |  |
| **Reb C** | .501^**^ | .788^**^ | .381^**^ |  |  |  |  |  |  |
| **Reb D** | -0.003 | -.300^**^ | 0.031 | -.154^**^ |  |  |  |  |  |
| **Reb E** | .567^**^ | -.481^**^ | -.126^**^ | -.115^**^ | .425^**^ |  |  |  |  |
| **Reb M** | -.683^**^ | -0.058 | 0.064 | -.437^**^ | .393^**^ | -.202^**^ |  |  |  |
| **Reb N** | .320^**^ | -.140^**^ | -.258^**^ | -0.011 | .464^**^ | .367^**^ | -0.005 |  |  |
| **Reb O** | -.409^**^ | -.217^**^ | -.164^**^ | -.468^**^ | .414^**^ | 0.035 | .650^**^ | .550^**^ |  |
| **TSG** | .483^**^ | .764^**^ | .546^**^ | .936^**^ | 0.019 | -0.006 | -.271^**^ | .102^*^ | -.303^**^ |

**Supplementary Table S3.** Haplotype means for QTL significant across multiple environments for maternal (10-RJR) haplotypes ‘a’ (Hap ‘a’) and ‘b’ (Hap ‘b’) and paternal (10-19) haplotypes ‘c’ (Hap ‘c’) and ‘d’ (Hap ‘d’) for several steviol glycoside traits in the F_1_ MSU 18-02 population.

|  |  |  |  |  |  | **10-RJR** | | **10-19** | |
| --- | --- | --- | --- | --- | --- | --- | --- | --- | --- |
| **Trait** | **QTL** | **Environment** | **LG** | **Position** | **Locus** | **Hap 'a'** | **Hap 'b'** | **Hap 'c'** | **Hap 'd'** |
| Stevioside | ***qST1.1*** | FVSU 2020 | 1 | 20.966 | Chr1_6937018 | 46.99 | 30.97 | 38.84 | 39.12 |
| Stevioside |  | SWMREC 2020 | 1 | 20.966 | Chr1_6937018 | 34.20 | 17.82 | 24.57 | 27.46 |
| Stevioside |  | HTRC 2020 | 1 | 20.966 | Chr1_6937018 | 31.55 | 15.77 | 22.75 | 24.57 |
| Stevioside |  | SWMREC 2021 | 1 | 20.966 | Chr1_6937018 | 32.55 | 20.64 | 25.67 | 27.52 |
| Reb A | ***qRA1.1*** | FVSU 2020 | 1 | 19.044 | Chr1_8842531 | 136.89 | 163.11 | 147.65 | 152.35 |
| Reb A |  | SWMREC 2020 | 1 | 15.929 | Chr1_8439641 | 142.31 | 172.16 | 152.68 | 161.80 |
| Reb A |  | HTRC 2020 | 1 | 5.863 | Chr1_2228998 | 103.56 | 122.99 | 113.21 | 113.34 |
| Reb A |  | SWMREC 2021 | 1 | 42.254 | Chr1_13460793 | 79.81 | 95.74 | 87.61 | 87.94 |
| Reb A |  | HTRC 2021 | 1 | 20.966 | Chr1_6937018 | 71.79 | 85.23 | 78.60 | 78.42 |
| Reb B | ***qRB1.1*** | HTRC 2020 | 1 | 19.044 | Chr1_8842531 | 3.67 | 5.66 | 4.57 | 4.76 |
| Reb B |  | SWMREC 2021 | 1 | 20.966 | Chr1_6937018 | 7.71 | 12.07 | 9.08 | 10.70 |
| Reb B |  | HTRC 2021 | 1 | 20.966 | Chr1_6937018 | 11.24 | 14.60 | 11.67 | 14.17 |
| Reb D | ***qRD1.1*** | SWMREC 2020 | 1 | 20.966 | Chr1_6937018 | 11.89 | 6.96 | 9.34 | 9.51 |
| Reb D |  | HTRC 2020 | 1 | 20.966 | Chr1_6937018 | 7.88 | 4.15 | 5.84 | 6.19 |
| Reb D |  | SWMREC 2021 | 1 | 20.966 | Chr1_6937018 | 11.74 | 6.89 | 8.88 | 9.74 |
| Reb D |  | HTRC 2021 | 1 | 20.966 | Chr1_6937018 | 9.91 | 5.27 | 7.08 | 8.09 |
| Reb E | ***qRE1.1*** | FVSU 2020 | 1 | 20.966 | Chr1_6937018 | 1.17 | 0.42 | 0.79 | 0.80 |
| Reb E |  | SWMREC 2020 | 1 | 20.966 | Chr1_6937018 | 1.24 | 0.27 | 0.73 | 0.78 |
| Reb E |  | HTRC 2020 | 1 | 20.966 | Chr1_6937018 | 0.97 | 0.23 | 0.57 | 0.63 |
| Reb N | ***qRN1.1*** | SWMREC 2020 | 1 | 20.966 | Chr1_6937018 | 3.93 | 2.19 | 3.01 | 3.11 |
| Reb N |  | HTRC 2020 | 1 | 20.966 | Chr1_6937018 | 2.42 | 1.43 | 1.84 | 2.00 |
| Reb N |  | SWMREC 2021 | 1 | 20.966 | Chr1_6937018 | 2.77 | 1.91 | 2.30 | 2.38 |
| Reb N |  | HTRC 2021 | 1 | 20.966 | Chr1_6937018 | 2.47 | 1.52 | 2.12 | 1.87 |

**Supplementary Figure S1.** Full genetic linkage map for the stevia population MSU18-02.

**Total 1322 markers on 11 linkage groups.**

**LG1: 152 markers LG2: 209 markers**

**LG3: 122 markers**

**LG4: 67 markers LG5: 89 markers**

**LG6: 171 markers LG7: 145 markers**

**LG8: 18 markers**


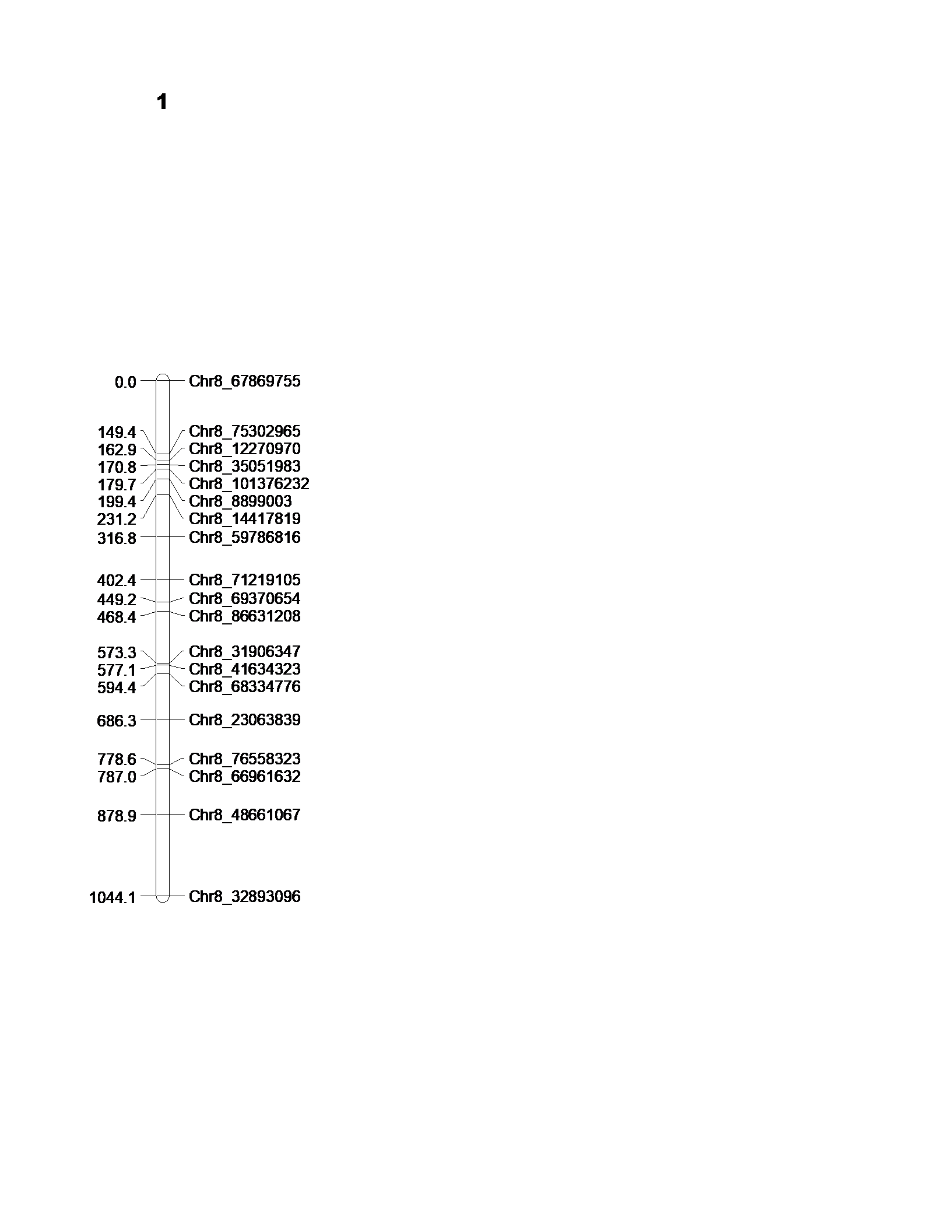


**LG9: 127 markers LG10: 108 markers**

**LG11: 114 markers**

**Supplementary Figure S2.** Comparison of 11 linkage groups with eleven stevia chromosomes (Xu et al., 2021) by AllMaps. Figure on left represents comparison of genetic positions (cM) of each linkage group with the physical positions (Mb) of corresponding chromosomes by straight lines. Figure on right represents the same comparison by dotted plot.

Supplementary Figure S2 (cont’d)

Supplementary Figure S2 (cont’d)

Supplementary Figure S2 (cont’d)

Supplementary Figure S2 (cont’d)

Supplementary Figure S2 (cont’d)

**Supplementary Figure S3**. Population distributions for concentrations of the steviol glycoside stevioside (a), rebaudioside A (b), rebaudioside B (c), rebaudioside C (d), rebaudioside D (e), rebaudioside M (f), rebaudioside E (g), rebaudioside N (h), rebaudioside O (i), and total steviol glycosides (j) for the stevia MSU18-02 population at HRTC 2020.


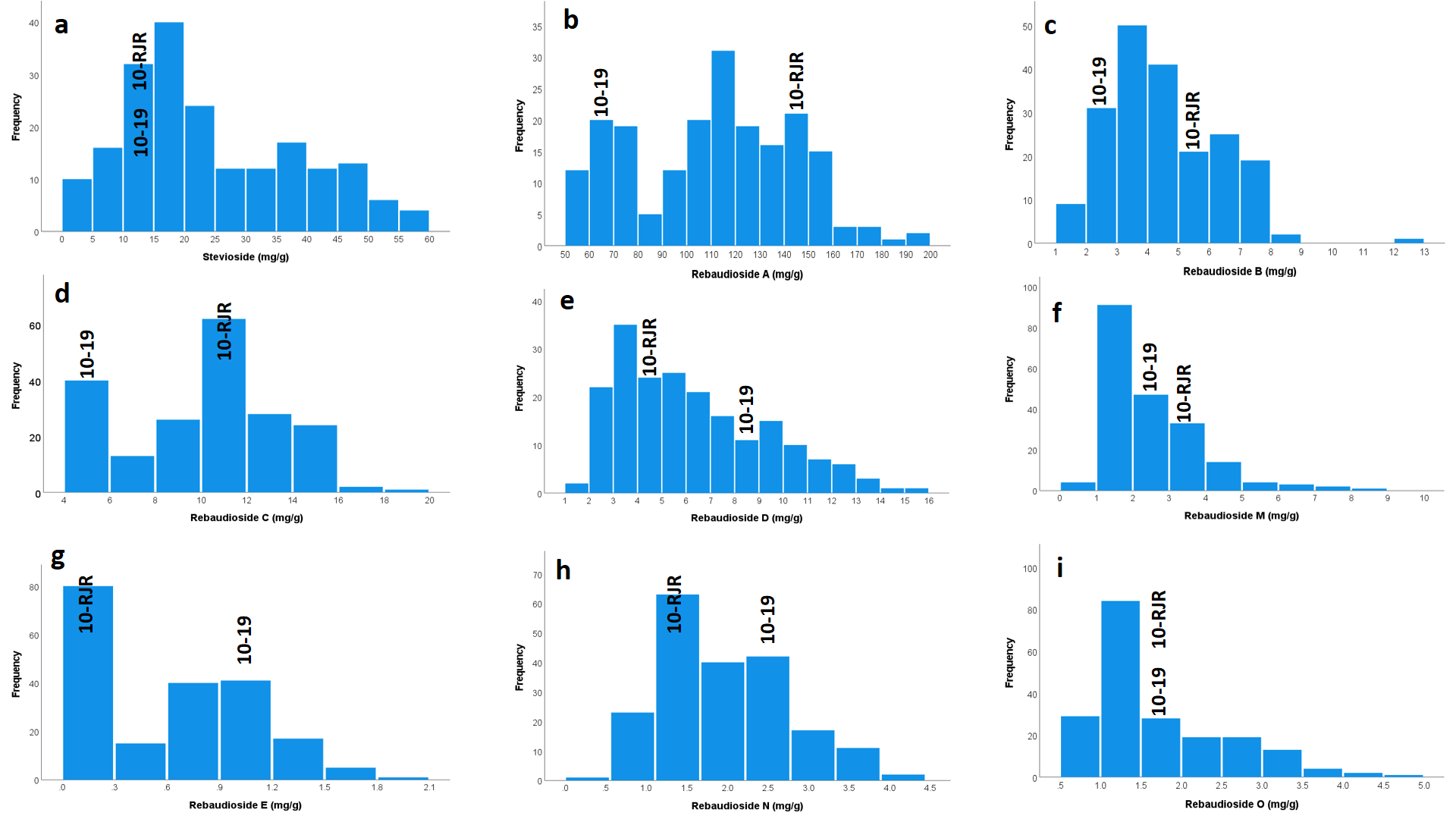


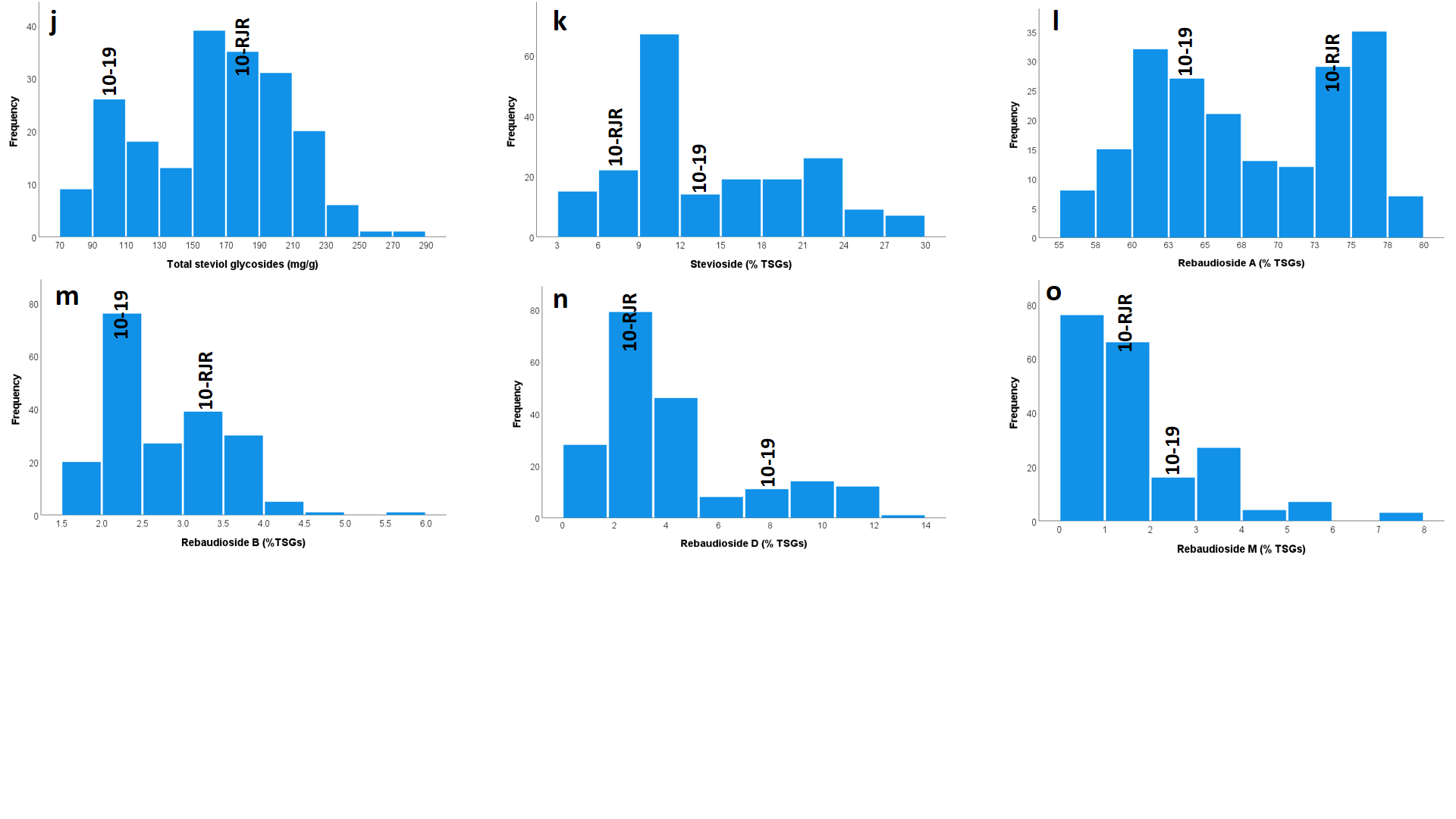


**Supplementary Figure S4**. Population distributions for concentrations of the steviol glycoside stevioside (a), rebaudioside A (b), rebaudioside B (c), rebaudioside C (d), rebaudioside D (e), rebaudioside M (f), rebaudioside E (g), rebaudioside N (h), rebaudioside O (i), and total steviol glycosides (j) for the stevia MSU18-02 population at SWMREC 2020.


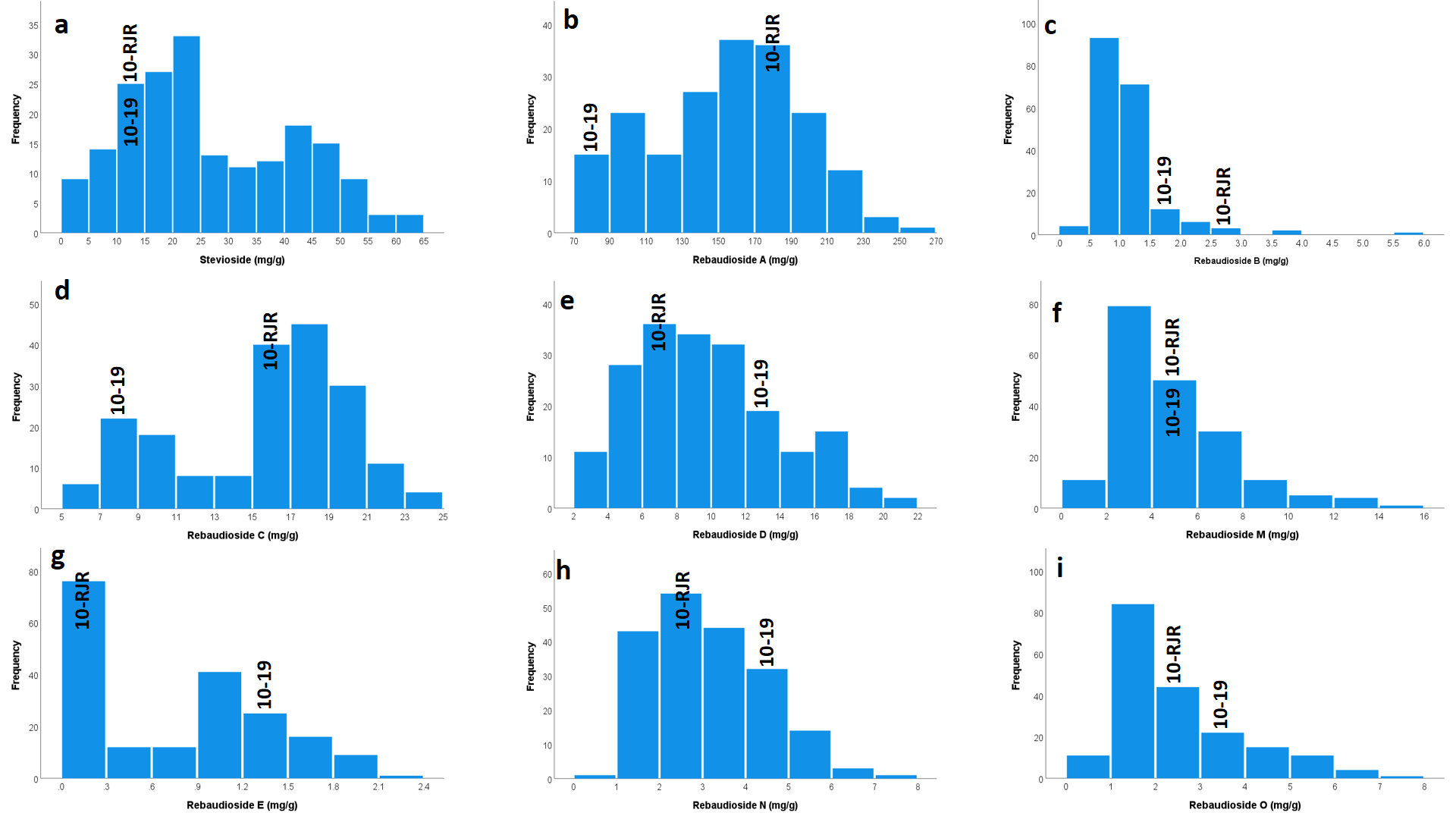


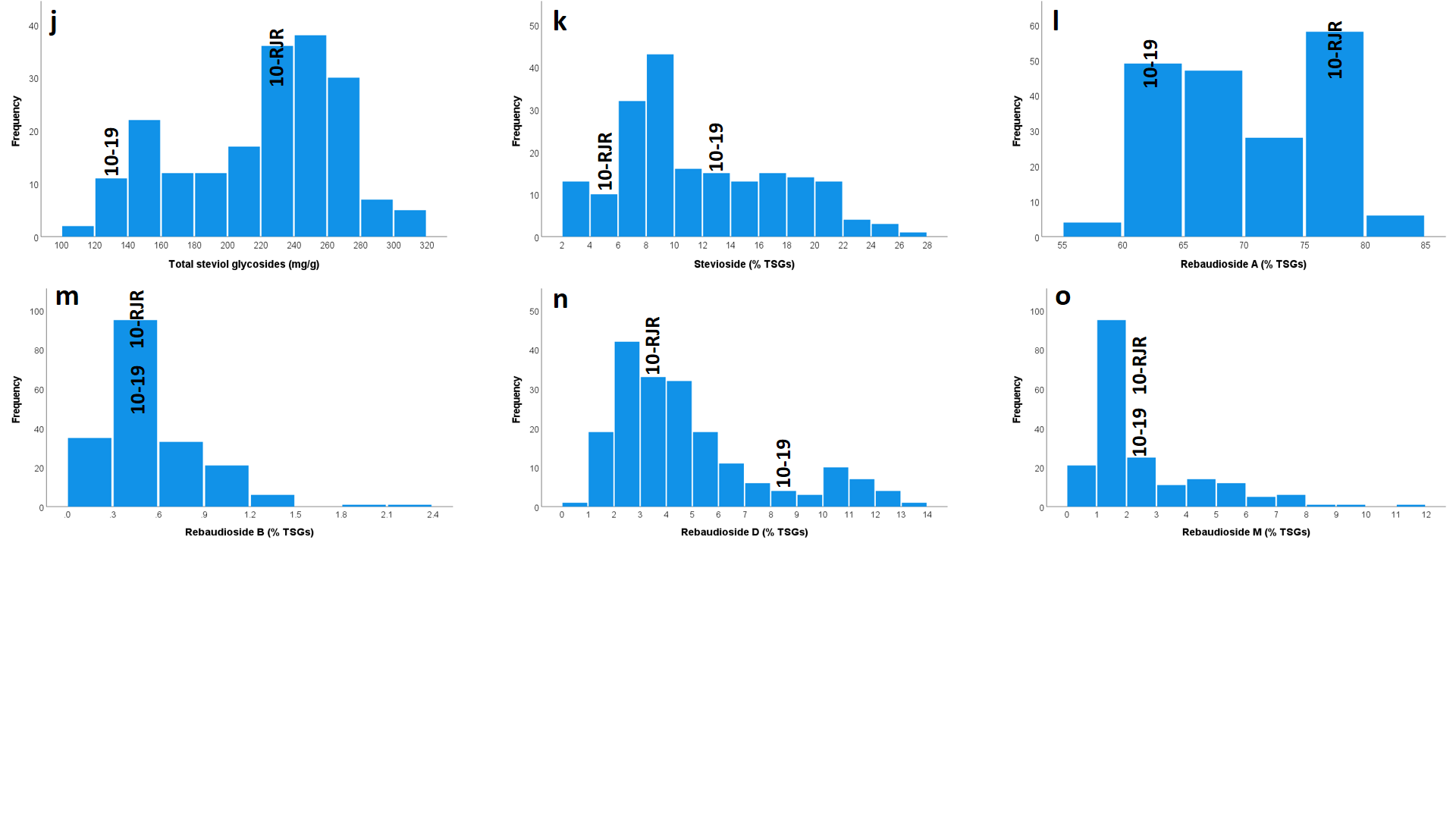


**Supplementary Figure S5.** Population distributions for concentrations of the steviol glycoside stevioside (a), rebaudioside A (b), rebaudioside B (c), rebaudioside C (d), rebaudioside D (e), rebaudioside M (f), rebaudioside E (g), rebaudioside N (h), rebaudioside O (i), and total steviol glycosides (j) for the stevia MSU18-02 population at FVSU 2020.


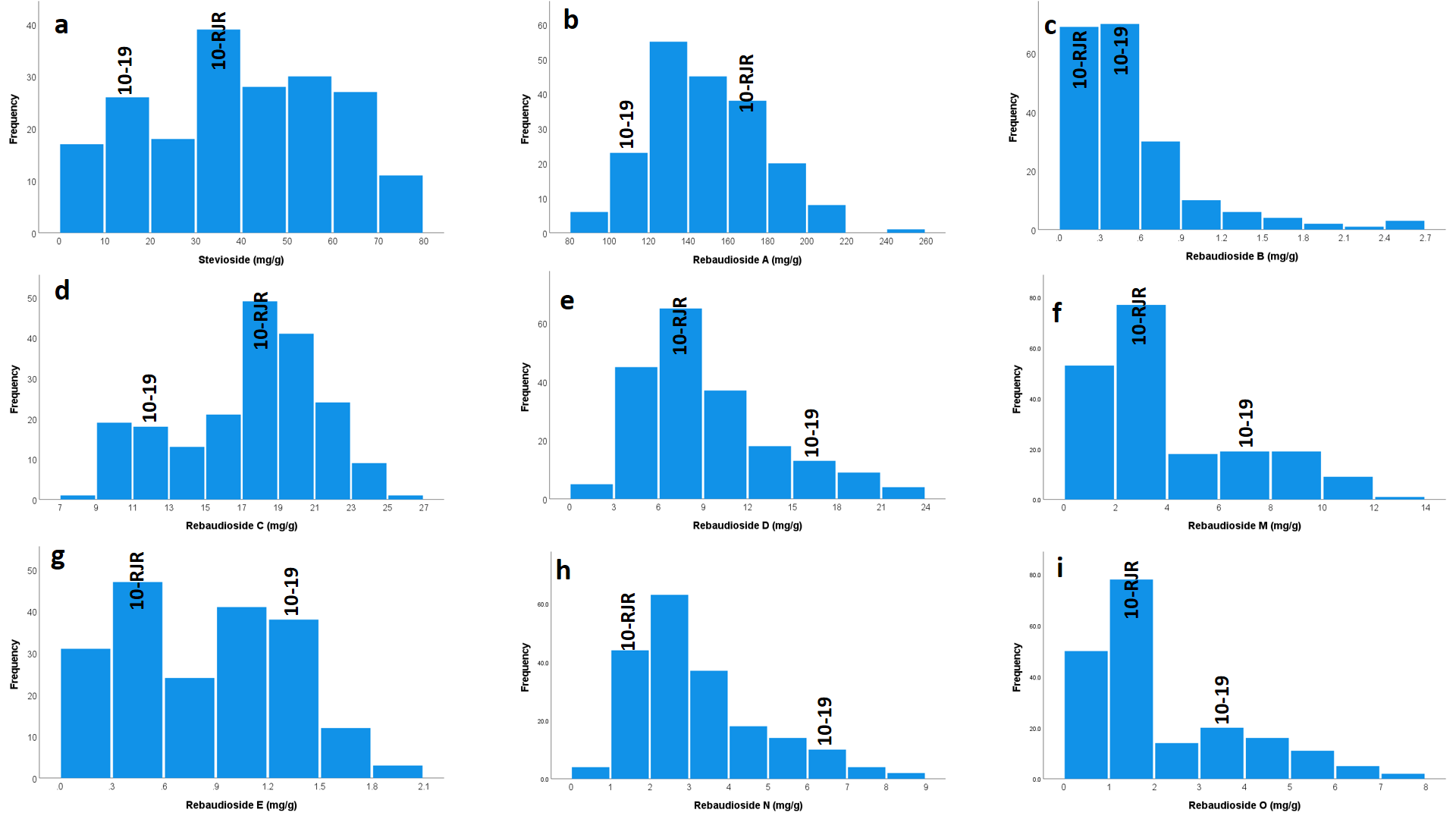


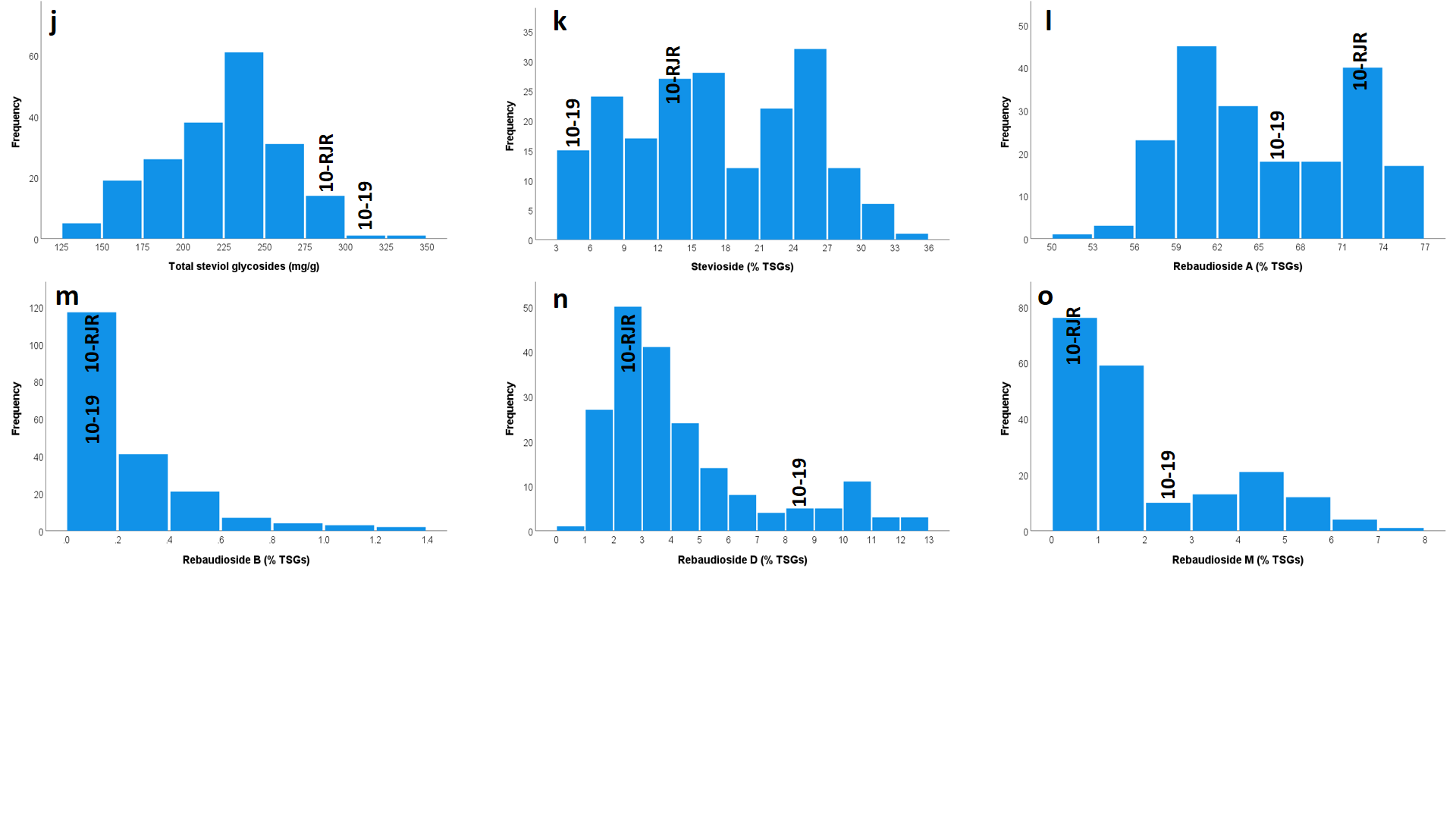


**Supplementary Figure S6.** Population distributions for concentrations of the steviol glycoside stevioside (a), rebaudioside A (b), rebaudioside B (c), rebaudioside C (d), rebaudioside D (e), rebaudioside M (f), rebaudioside E (g), rebaudioside N (h), rebaudioside O (i), and total steviol glycosides (j) for the stevia MSU18-02 population at HTRC 2021.


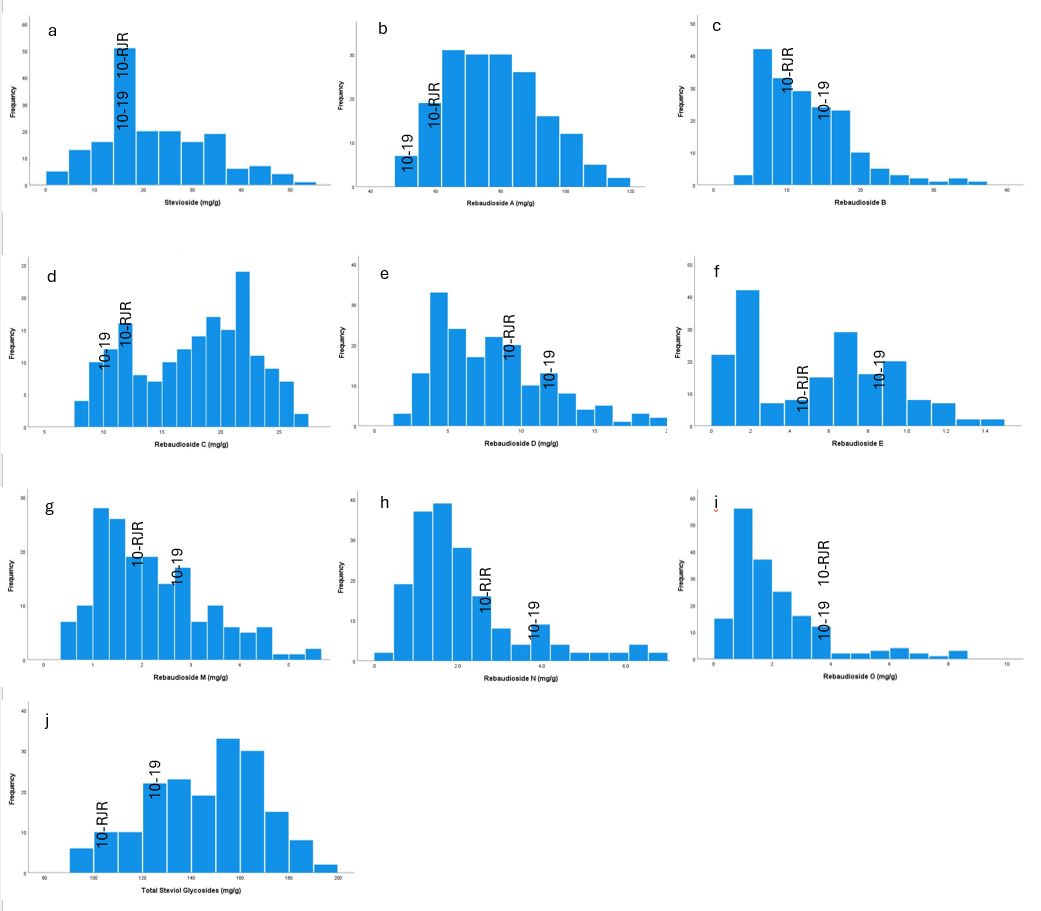


**Supplementary Figure S7.** Population distributions for concentrations of the steviol glycoside stevioside (a), rebaudioside A (b), rebaudioside B (c), rebaudioside C (d), rebaudioside D (e), rebaudioside M (f), rebaudioside E (g), rebaudioside N (h), rebaudioside O (i), and total steviol glycosides (j) for the stevia MSU18-02 population at SWMREC 2021.


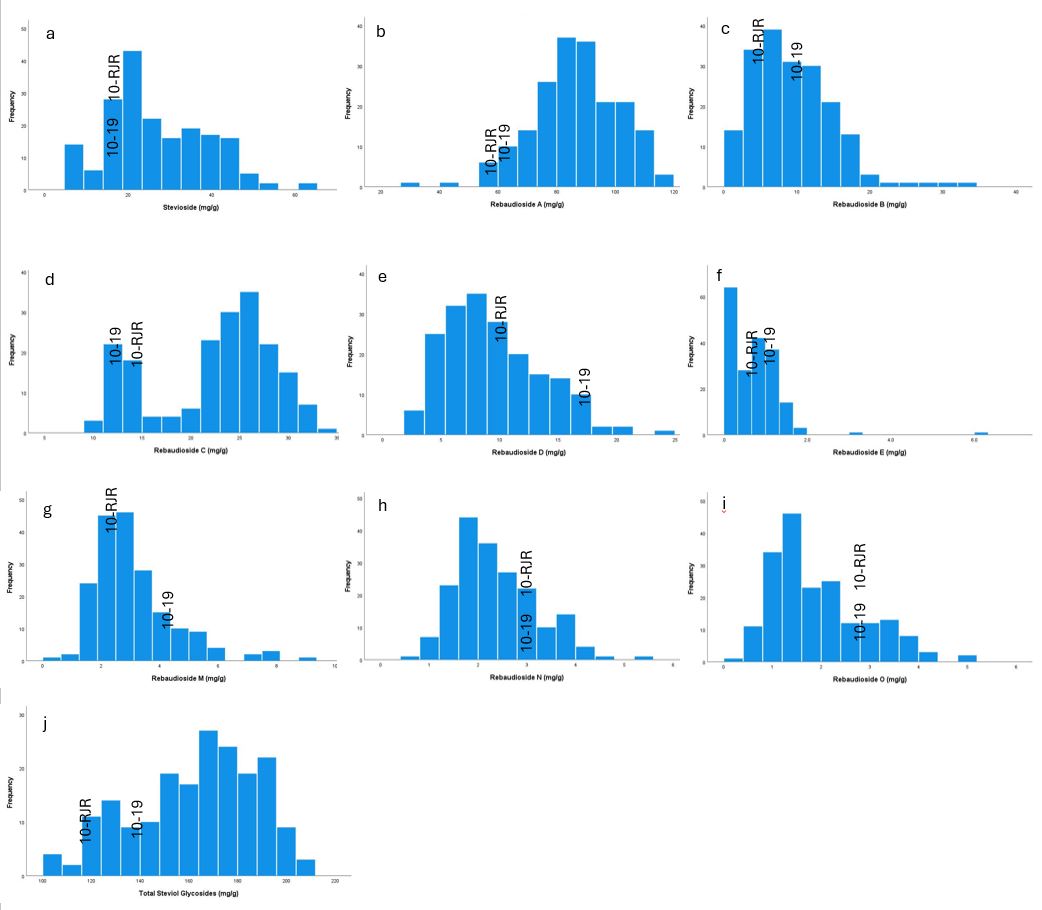


**Supplementary Figure S8**. LOD score profiles for chromosomes harboring significant QTL for a) stevioside, b) Reb A, c) Reb B, d) Reb C, e) Reb D, f) Reb E, g) Reb N, and h) total steviol glycosides.

1. Stevioside


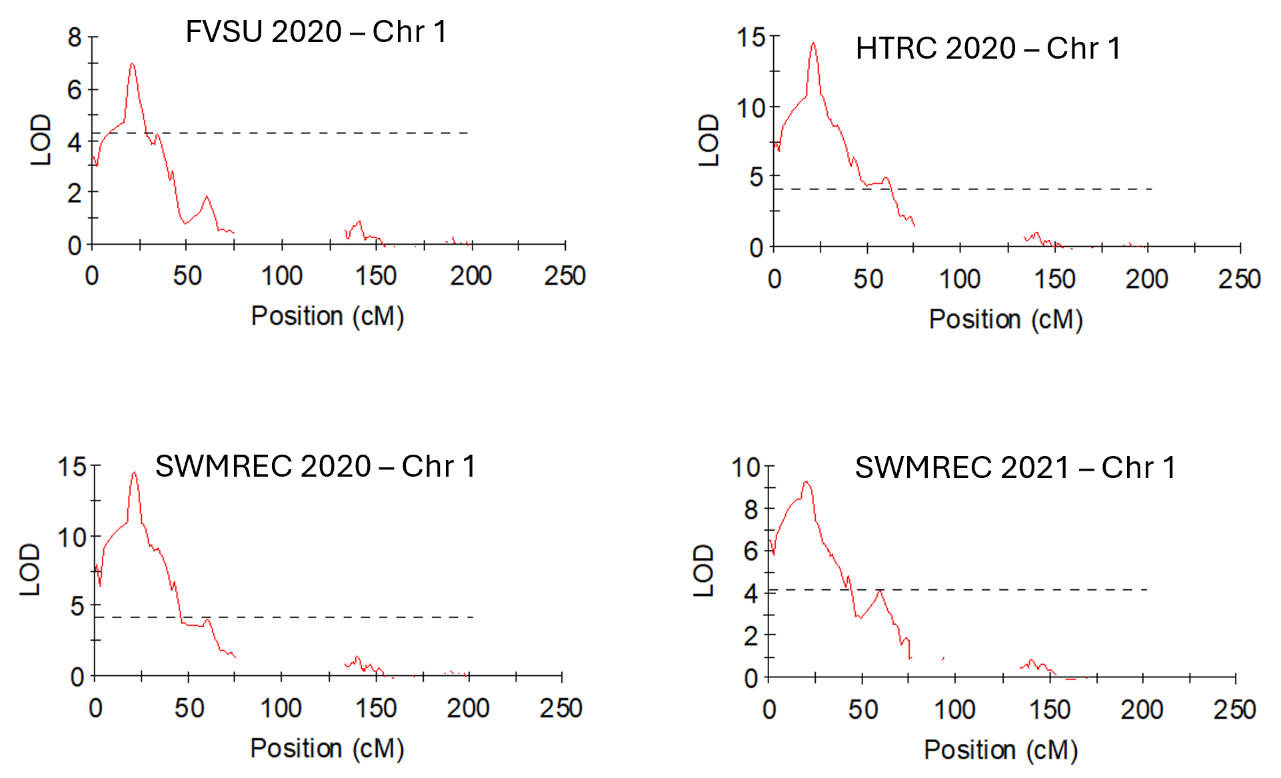


Fig. S8 (cont’d)

1. Reb A


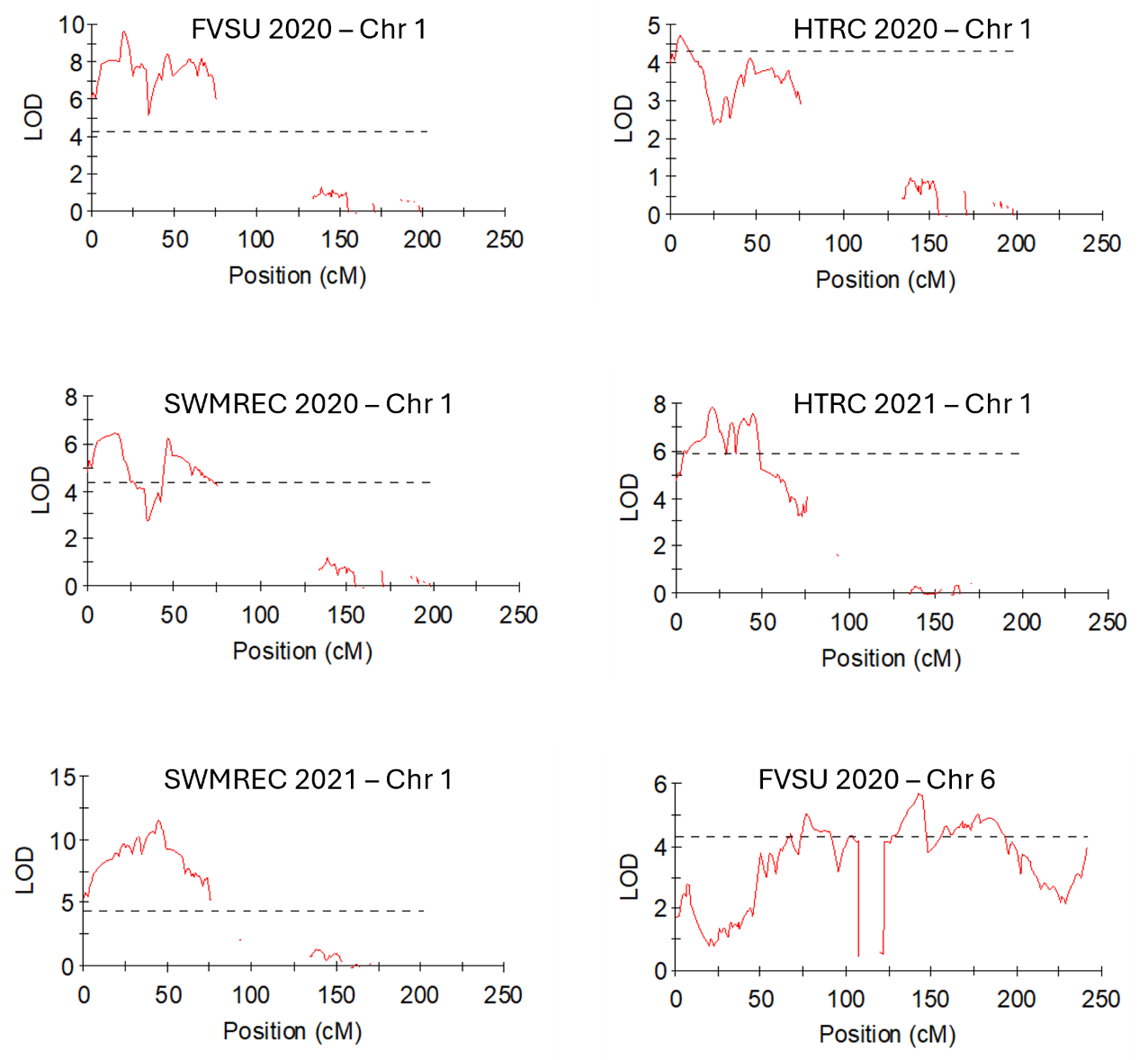


Fig. S8 (cont’d)

1. Reb B


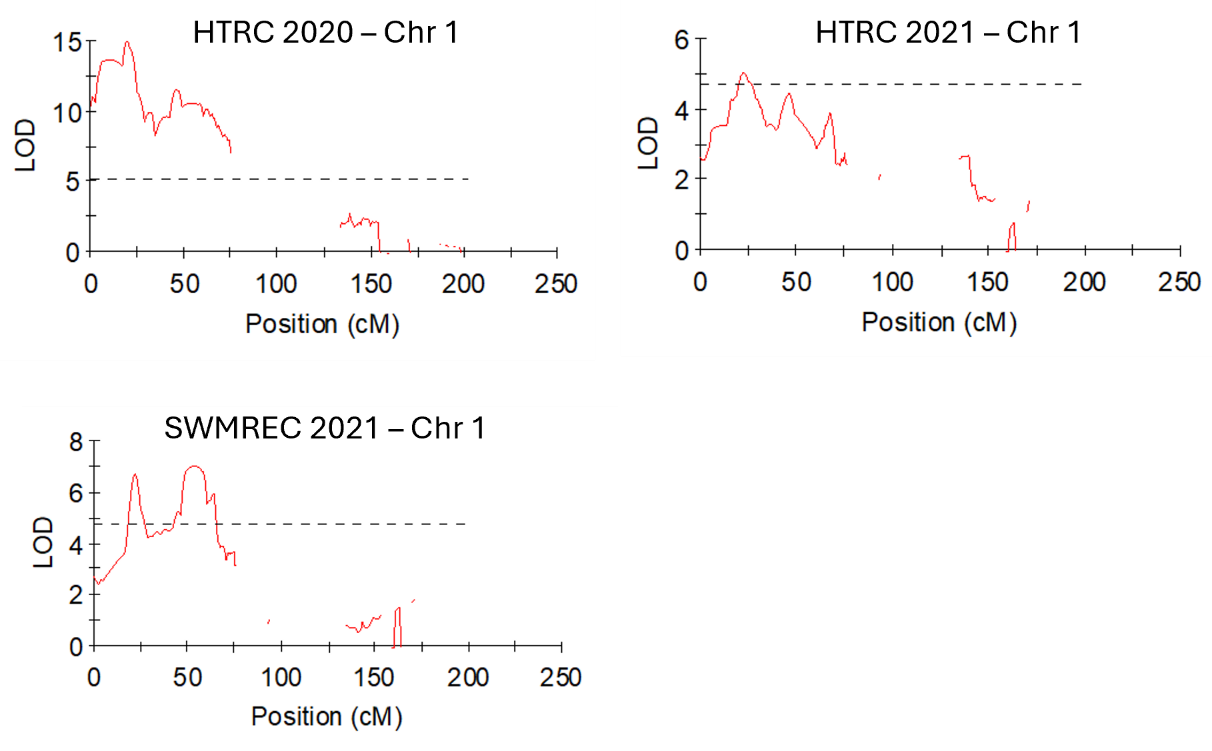


1. Reb C


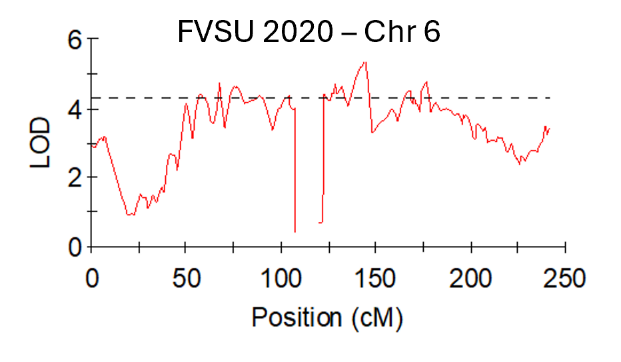


Fig. S8 (cont’d)

1. Reb D


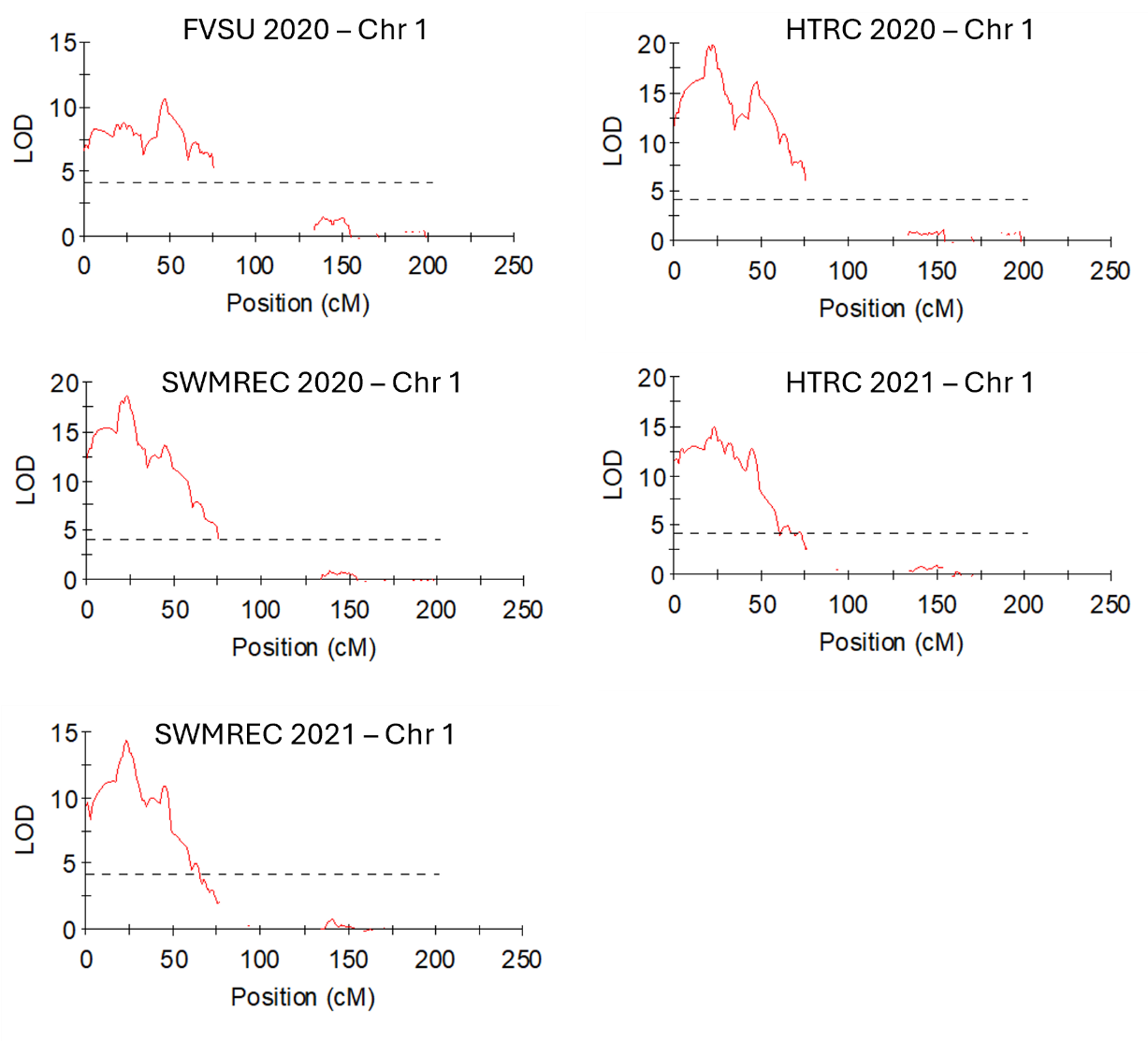


Fig. S8 (cont’d)

1. Reb E


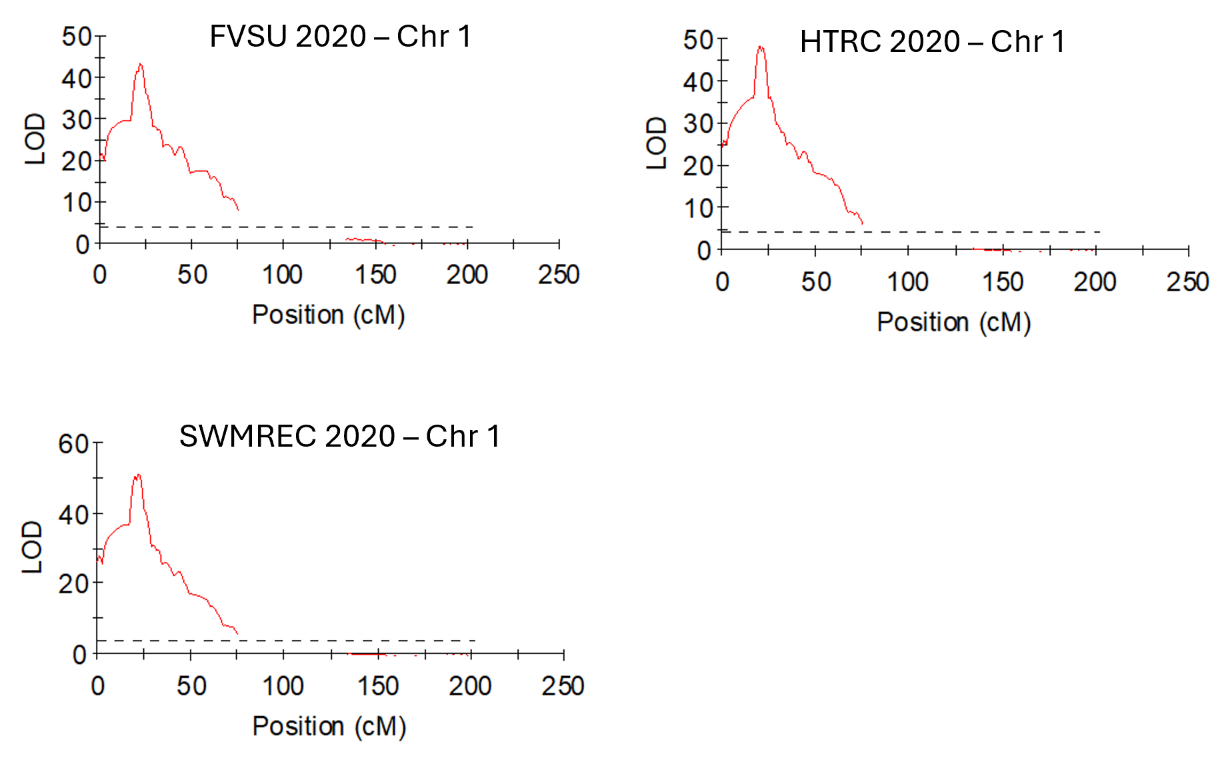


Fig. S8 (cont’d)

1. Reb N


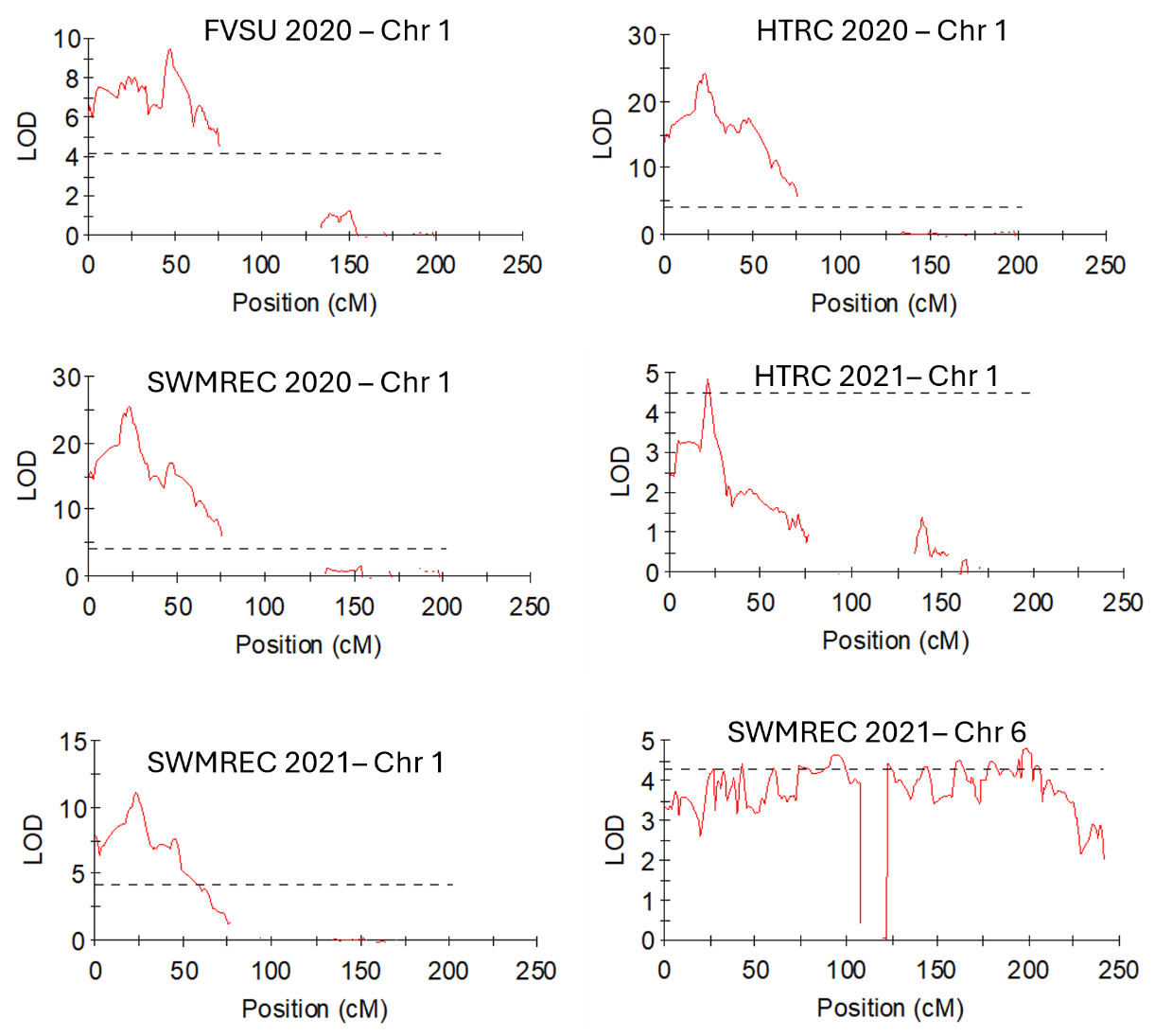


1. Total steviol glycosides


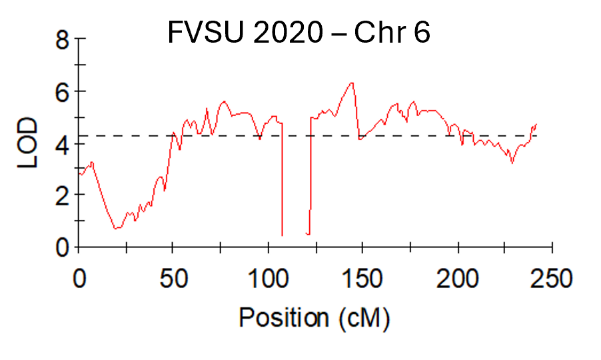


Supplementary Figure S9. Recombination fraction matrices for (A) the full stevia linkage map and (B) a closeup of linkage group 8; (C) LOD plot for linkage group 8.


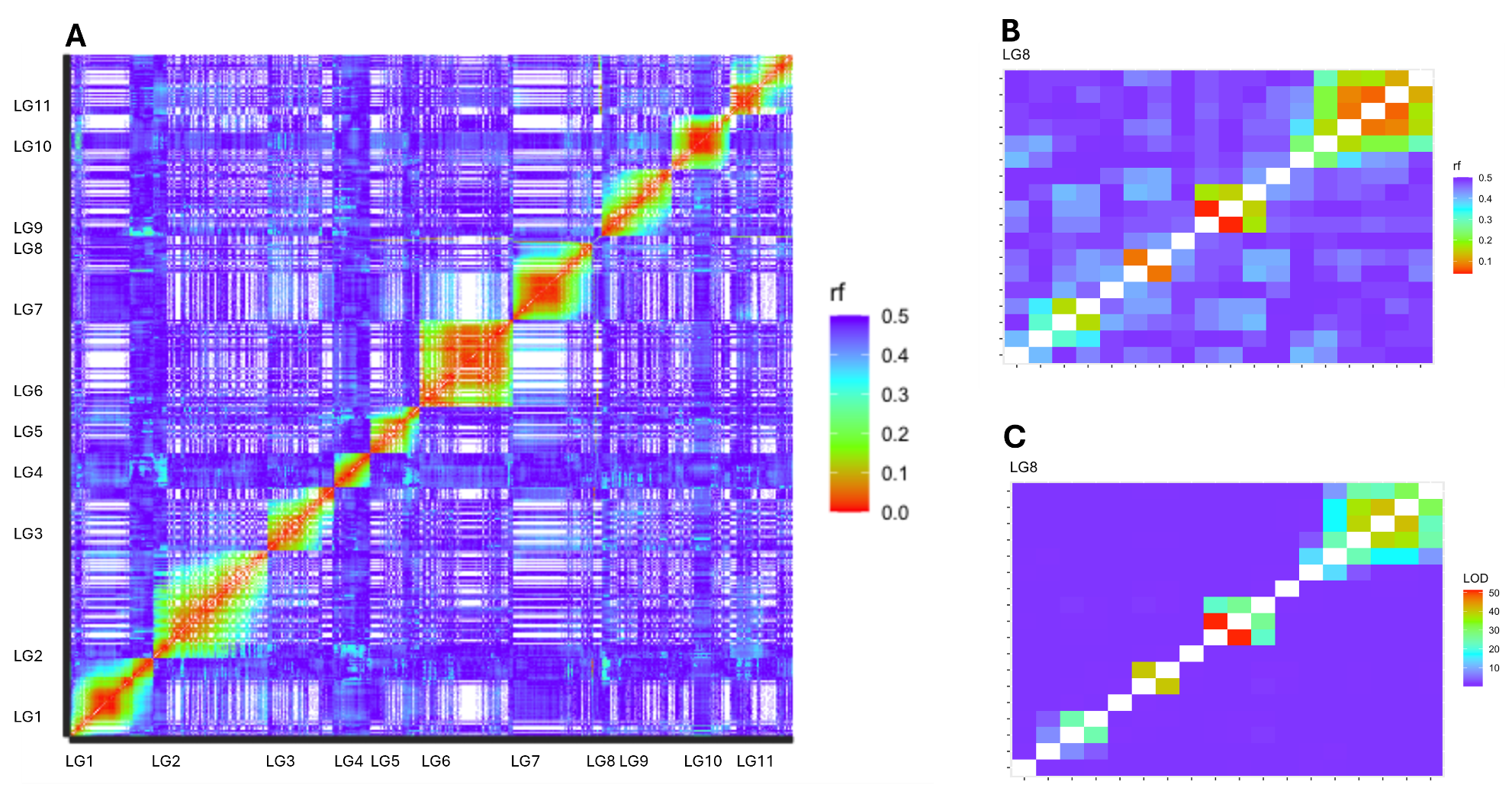

Supplement: jkag015_Supplementary_Data [file jkag015_supplementary_data.docx]
